# Interpreting and de-noising genetically engineered barcodes in a DNA virus

**DOI:** 10.1371/journal.pcbi.1010131

**Published:** 2022-11-22

**Authors:** Sylvain Blois, Benjamin M. Goetz, James J. Bull, Christopher S. Sullivan

**Affiliations:** 1 Department of Molecular Biosciences, LaMontagne Center for Infectious Disease, The University of Texas at Austin, Austin, Texas, United States of America; 2 Department of Biomedical Sciences, University of Cagliari, Monserrato, Cagliari, Italy; 3 Center for Biomedical Research Support, The University of Texas at Austin, Austin, Texas, United States of America; 4 Department of Biological Sciences, University of Idaho, Moscow, Idaho, United States of America; ETH Zurich, SWITZERLAND

## Abstract

The concept of a nucleic acid barcode applied to pathogen genomes is easy to grasp and the many possible uses are straightforward. But implementation may not be easy, especially when growing through multiple generations or assaying the pathogen long-term. The potential problems include: the barcode might alter fitness, the barcode may accumulate mutations, and construction of the marked pathogens may result in unintended barcodes that are not as designed. Here, we generate approximately 5,000 randomized barcodes in the genome of the prototypic small DNA virus murine polyomavirus. We describe the challenges faced with interpreting the barcode sequences obtained from the library. Our Illumina NextSeq sequencing recalled much greater variation in barcode sequencing reads than the expected 5,000 barcodes–necessarily stemming from the Illumina library processing and sequencing error. Using data from defined control virus genomes cloned into plasmid backbones we develop a vetted post-sequencing method to cluster the erroneous reads around the true virus genome barcodes. These findings may foreshadow problems with randomized barcodes in other microbial systems and provide a useful approach for future work utilizing nucleic acid barcoded pathogens.

## Author summary

The use of randomized barcodes in microbial genomes can be a powerful technique for the study of microbes, including for understanding intrahost dynamics of infection. Here we provide a vetted approach for interpreting barcode repertoire that was developed using defined control barcode genomes of the model small DNA virus murine polyomavirus and applied to the derivative virus library and intermediate libraries that went into the generation of virus stock. We present a series of experimental results and observations that may assist others in the design and application of barcodes to their microbe of interest.

## Introduction

The within-host population dynamics of a microbe are usually studied as population abundances across time and tissues [1,2]. Although informative, this approach is blind to differences among individuals within populations. Thus, a virus concentration of 10,000 per mL maintained over time could be achieved if 10,000 lineages are all just maintaining themselves or could alternatively be achieved if 100 lineages are each producing 100 progeny and the other 9,900 are cleared by the host. Knowing those dynamics can shed light on the different processes that may be occurring during infection (e.g., latency of part of the population) and can reveal the extent of tissue subdivision of the infection and population bottlenecks. An understanding of dynamics may even inform treatment alternatives.

Technical advances in genetic engineering and sequencing now allow microbial populations to be established in which each individual microbe can be distinguished from nearly all others in an inoculum even though all individuals are the same genetic strain. With this technology, known as DNA barcoding, it becomes possible to create separate identities for potentially millions of individual lineages used to simultaneously infect a single host [3–10]. Barcoding usually involves the insertion of short, randomized DNA segments in the genome, the number of different types increasing as 4^N^, where N is the number of bases in the randomized insert. Each lineage is then defined by its unique barcode, and all descendants from each infecting individual can be discriminated from descendants of other infecting individuals through inheritance of the barcode. In essence, barcoding enables a pedigree analysis of an ongoing population.

The analysis of barcodes is trivial when they do not change from parent to offspring. But barcodes may be prone to mutation and sequencing errors. In that case, analysis of ancestor-descendant relationships will require a statistical correction (clustering) to group non-identical barcodes that nonetheless share common ancestry. The suitability of any specific clustering algorithm may depend on the biological details of the implementation [11]–the various factors contributing to parent-offspring differences in barcode sequence as well as the extent of differences among the barcodes in the founding population. But the clustering principles should transcend specific applications.

**Fig 1:**
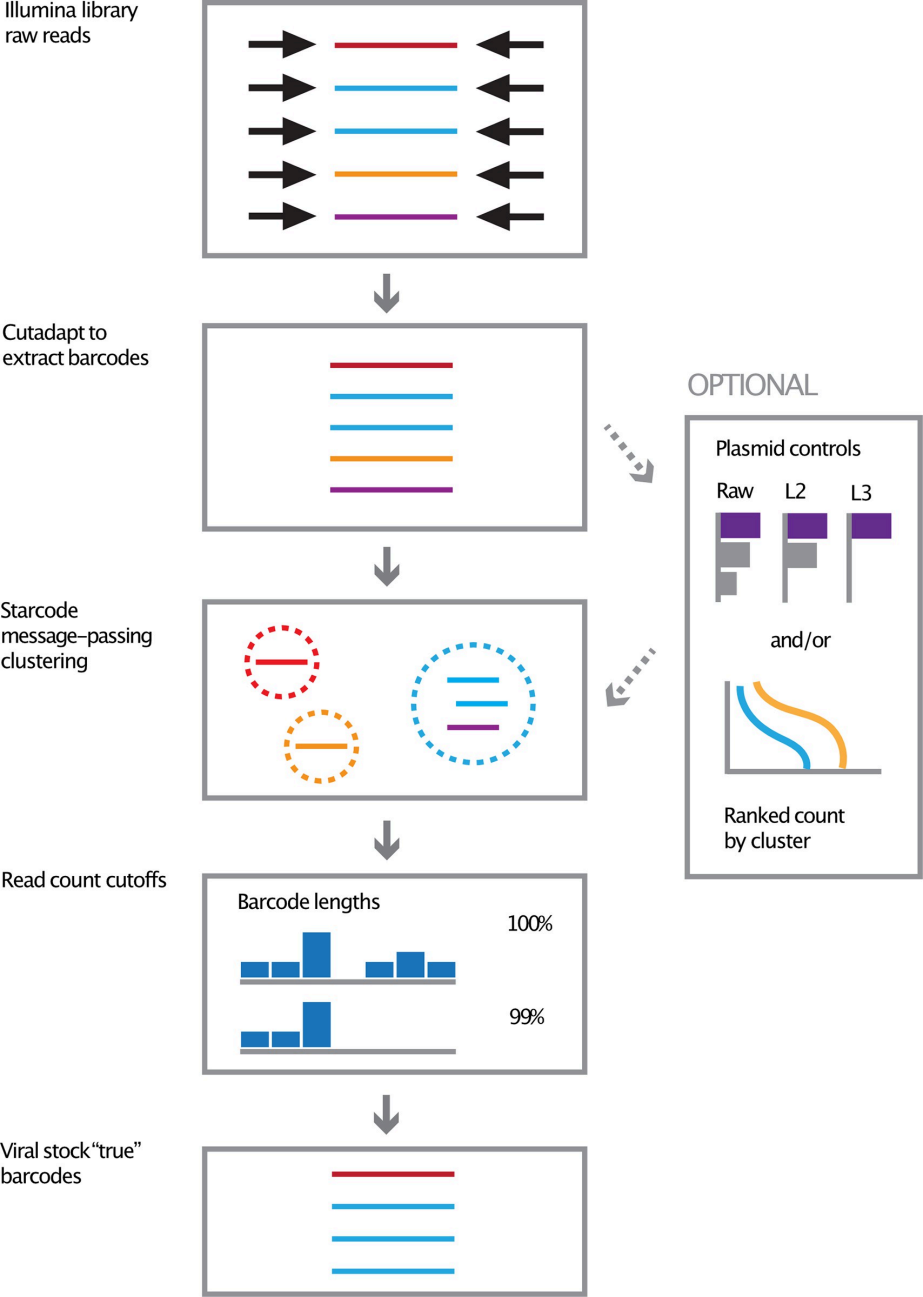
Outline of computational workflow for selecting stock barcodes. Linker sequences are trimmed with Cutadapt (colors represent distinct barcodes). Clustering is performed using the Starcode message-passing algorithm to determine centroids, defining "centroid" as the representative barcode (dotted circles represent clusters). We empirically determined which edit distance to employ by examining two criteria: 1) the performance of different edit distances on various amounts of plasmid controls, each containing a barcode of known sequence, to recall correct input barcode sequences and 2) “shoulder cutoff” using the plot line of the distribution abundance of called clusters from our barcoded plasmid library. In defining the "true" barcodes of our virus library, we utilized L = 3 Levenshtein distance because it performed slightly better in our defined barcode plasmid controls over the default Starcode Levenshtein distance of L = 2. Final "true" barcodes were then assigned by omitting the lowest abundance barcodes (omitting the bottom barcodes that account for a cumulative 1% of all counts), based on the assumption that the lowest abundance reads were most likely to be sequencing artifacts.

Here, we describe the creation of barcoded polyomaviruses that were generated from a library of barcoded plasmids (Figs 1 and S1, S2, S3). When the sequences of the plasmid library were analyzed, it became obvious that a substantial fraction of sequencing reads had to be errors. We compared various correction strategies including eliminating lower confidence reads, assigning an abundance cutoff, and applying a vetted barcode clustering algorithm under various conditions to associate the erroneous barcode sequences with the true parent barcode. We utilized a series of defined experimental known barcode genome controls and simulations to empirically ascertain appropriate clustering parameters. This system afforded considerable control over and investigation of the factors that complicate the interpretation of barcode abundance with clustering proving essential to the interpretation of barcodes and their abundances. We characterize the virus library and the components that went into individual steps of its construction. The result is an in depth understanding of different approaches for error correction and the functionality of different computational clustering parameters. These findings provide a functional approach applicable to experimental infections with murine polyomavirus (muPyV) and likely other barcoded microbes.

## Results

### Engineering barcoded DNA virus genomes as plasmids

In brief, we engineered the murine polyomavirus (muPyV) (dsDNA genomes of ~5.4 kbp) to carry 18 bp inserts, 12 bases of which were randomized A, C, G, T with equal probability. We chose the non-protein-coding genomic region in between the opposing early and late polyadenylation sites for the insert, which was suggested as a viable location based on the tolerance of Bandicoot Papillomatosis Carcinomatosis Virus 1 (BPCV1) to a large natural genomic insert in an analogous region and a restriction site being successfully engineered in a similar location in a previously described murine PyV mutant [12–14]. We cloned barcodes into bacterial plasmids containing the virus genome and then generated barcoded viruses as described in Methods. Barcoded virus stocks gave rise to high titers of virus (the virus library concentration gave rise to 2.50 x 10^6^ infectious units per mL compared to 7.14 x 10^6^ for wild-type, as determined by immunofluorescence assay), implying no overt fitness defects of these barcoded viruses.

### Maximally ~5,700 unique barcodes

We estimated the size of the plasmid library that gave rise to the virus library to be 5,678 total bacterial colonies. Since all viruses emanated from the plasmid library, this number is necessarily the maximum possible number of unique barcodes present in both the plasmid and virus libraries. However, this is an upper limit and also does not mean that all barcodes are equally represented at the level of copies per barcode.

To get a sense of barcode similarity among viruses, the barcodes of 21 separate clones were Sanger-sequenced individually, as described in Methods; 20 were unique. At first blush, this approach seems to be a reasonable test of barcode depth in the virus library, and indeed, if a major part of the virus library had been dominated by one barcode, this test should have detected it. But the 20/21 is potentially misleading if interpreted as if 95% of the clones were unique. The test here is similar to the well-known ‘birthday paradox’ in which the question is posed as to the smallest group size in which the odds that at least 2 people share a birthday is at least 0.5. The answer to the ‘birthday paradox’ is a group size of 23. From this perspective, the observation of 20 unique barcodes in a sample of 21 might be compatible with a much smaller library size than 5,678 or be compatible with strong skews in the relative abundance of the different types. A direct numerical analysis of the problem (the number of single matches in 21 random draws from a population of unique individuals with replacement) indicates that there is only a 5% chance of observing a single match if the population size is approximately 4,000 equally represented types. Furthermore, the outcome of a single match in a sample of 21 is more often observed with a population size of 200–300 than with larger population sizes, suggesting that 5,700 is a vast overestimate of our library size. This also reveals that 20/21 unique barcodes is not a sensitive metric for evaluating library size. Nonetheless, other evidence presented below is consistent with a number of unique barcodes close to 5,000. One reconciliation of these seemingly disparate observations is that the different barcodes are not equally abundant in the plasmid library, a possibility that will be supported by other subsequent observations.

Regardless of the exact number of unique barcodes, it is clear that the barcoding was somewhat sparse in the space of 4^12^ possibilities, with at least 2,940 barcode possibilities for every barcode present (4^12^ /5,678). As will be developed below, this sparseness was critical in allowing us to discover–and resolve–errors in the assaying of barcodes. At the same time, even greater sparseness would have been desirable (as will be shown below).

### Illumina library sequences exhibit elevated variation in barcodes

lllumina NextSeq SR75 was used to sequence the virus library. In contrast to the expected maximum of ~5,700 barcodes, almost 100-fold more barcode sequences were observed (535,772 barcodes from 29,867,853 total reads). In addition, orders of magnitude variation existed in the abundances of the different barcode reads (these data will be presented and analyzed below).

This variation must have arisen after the founding of the ~5,700 bacterial colonies. The steps where this error could have been introduced include mutations during colony growth, virus production from the plasmids, Illumina library preparation, and Illumina sequencing itself. To narrow these possibilities, the plasmid library was sequenced; recall that the plasmid library was the immediate progenitor of the virus library. Similar to the virus library, approximatively 274,213 different barcodes were detected (out of 10, 765, 143 total reads) in the plasmid library, indicating that the virus production step was at best a minor contributor to the error (e.g., two-fold).

To gain insight to whether the errors were arising during growth of the plasmid, we resorted to a bioinformatic approach. The bacterial mutation predictor program (EFM calculator) applied to the 449 bases across the barcode indicated that the sequence was exceptionally stable, with base mutation rates no larger than 10^−10^ [15]. Thus, the biological sources of error appear unlikely. In contrast, errors from Illumina sequencing using 2-channel chemistry are known to be susceptible to nucleotide balance, so the sequencing platform is intrinsically suspect as the source of this error (https://support.illumina.com/bulletins/2016/07/what-is-nucleotide-diversity-and-why-is-it-important.html). Our design of staggered amplification primers attempted to avoid extreme nucleotide imbalances, so the source of error remains somewhat of an enigma.

High error in barcode sequences has been reported previously and thus seems to be a common problem [16]. Its source–whether biological or technical–is of little consequence for the analysis here, but understanding its origin may help reduce the problem in future studies.

### Possible resolutions of barcode error

With such large error rates, use of the raw barcodes would grossly confound interpretation of viral identities: the errors mean that multiple apparent barcodes are derived from the same parent, yet we neither know the true barcodes nor which barcodes belong to the same parent. However, appropriate correction of the barcode errors may mitigate the problem. Several error-reduction methods may be entertained.

1. Abundance cutoff: merely discard low-copy barcodes
2. Sequence quality standards: discard barcodes with low quality bases
3. Cluster: group barcodes that appear to originate from the same parent

Each of these methods is often applied in barcode studies without knowledge of the specific effects on the outcome. In subsequent sections, we evaluate each of these methods. All methods will be found to have merits and drawbacks. One of the main points of our study will be that the decision about which error correction method(s) to apply will vary with the application, that there is no universal solution. Choices should be made in light of the magnitude of errors present as well as properties of the library. The method best suited to our data may not the best for other applications, but the protocol outlined here should be useful in any application. Our goal is thus to illustrate how to evaluate the alternative error-reduction methods in context.

#### Abundance cutoff

This error correction method is straightforward. The underlying assumption is that errors are individually rare in the sequence reads, the true barcode being by far the most abundant. Thus, when the approximate number of true barcodes is known (say, N), the cutoff requires choosing the N most abundant barcodes in the sequence reads. For our data set, that would mean choosing the ~5,000 most abundant barcodes. This method should work best when all true barcodes are approximately equally abundant in the libraries, so that erroneous barcodes are invariably rarer than true barcodes.

#### Sequence quality standards

If errors arise in the sequencing reaction itself, erroneous reads may disproportionately contain low-quality base scores. If low quality scores apply to the different types of errors that can arise, error correction is then largely a matter of discarding individual barcode reads containing any base positions with low quality scores.

#### Clustering

This is the most complicated of the three error correction methods. For the case we consider, the assumption is that the errors in a barcode–whatever their source–are independent and moderately uncommon. In turn, this means that a parent barcode (the true one) will give rise to erroneous ‘progeny’ barcodes that are more similar to it than to other parent barcodes. As a simple example, a parent barcode ‘HONEY’ may have progeny of ‘MONEY’ and ‘HONED’ and ‘HOMEY.’ All of these progeny could be unambiguously assigned to the parent HONEY instead of, say, the parent barcode ‘FUZZY.’ From this example, it is easily seen that clustering can be successful only to the extent that the parent barcodes are sparse in the space of possible barcodes. That is, if HONEY, MONEY, HONED, and HOMEY were included among the parent barcodes, it would not be possible to distinguish parent barcodes from their erroneous progeny based on sequence alone. However, counts for each barcode provide additional information. Assuming a moderate error rate, progeny barcodes from a high-count parent barcode are more prevalent than progeny barcodes from a lower-count parent barcode. Thus, two barcodes that are similar in sequence and count are unlikely to stem from one being progeny of the other. Continuing the above example, a progeny barcode such as ‘HONED’ would more naturally be associated with a possible parent ‘MONEY’ than a possible parent ‘HONEY’, if the count for ‘MONEY’ is much higher than the count of ‘HONED’, and if the counts of ‘HONEY’ and ‘HONED’ are roughly the same.

As a clustering algorithm, we utilized Starcode which is particularly suited to nearly identical length sequences, with a significant range of counts [17]. For our initial implementation, we utilized Starcode’s message-passing algorithm (described below in the multiple-plasmid section). A crucial parameter for Starcode is the Levenshtein distance (L), which counts the number of simple ‘mutations’ (base changes, single base deletions, and single base insertions) required to render two sequences identical. The Levenshtein distance is never longer than, and is often shorter than the more commonly known Hamming distance (the latter merely counts differences between two sequences in the same reference frame). To compare Levenshtein and Hamming distances, consider the sequences ABCD and BCDA. The Hamming distance is 4 because each position is different. The Levenshtein distance is 2: an insertion of A at the start and deletion of the last base renders them the same.

For 12 nt sequences (our barcode length), Starcode’s default value for Levenshtein distance is L = 2, although there is little published evidence to justify which distance parameter is best for barcodes–and the appropriate distance will depend on the types and magnitudes of errors. We therefore test the effects of different L-values in our data.

### Single-plasmid controls: all three error corrections improve outcomes

An important step in deciding which correction methods to apply is to analyze the errors in controls–samples with known barcodes such that the magnitude and types of errors can be known with certainty. Indeed, the most appropriate error correction method cannot be decided without knowing something about the nature of the errors. We thus analyze controls.

We developed controls using plasmid DNA containing the barcoded virus genomes. Plasmid DNA has the advantages that (i) DNA can be isolated from a single colony and thus is known to be limited to one parental barcode, and (ii) that parental barcode can be determined as a consensus sequence using Sanger sequencing. It should be noted that the barcode in plasmid DNA exempts steps in virus creation and thus eliminates a step at which mutations may be introduced. Were the vast majority of errors introduced in virus creation (which is not the case here), plasmid barcode controls would be largely error free and thus not be useful in decisions about error correction methods.

**Fig 2:**
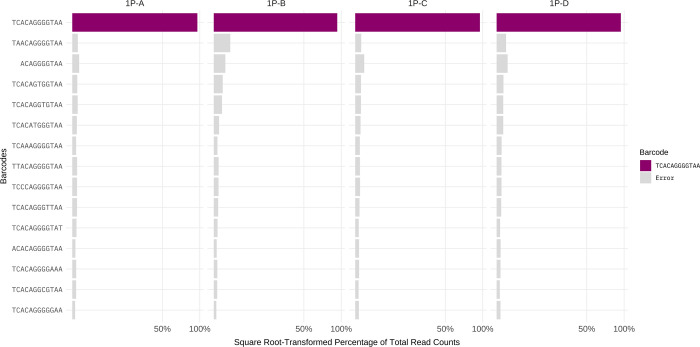
Illumina sequencing reads from single-plasmid controls. The control spiked-in barcode is shown as purple bars, erroneous barcodes are shown as gray bars. Shown are the top highest count 15 barcode sequences tallied across the four 1-Plasmid controls. Raw counts are shown with no clustering applied, with the x-axis square root-transformed to highlight low values.

Our controls used a single plasmid whose barcode region was sequenced with both Sanger and Illumina methods; alternative versions of this control varied the concentration of plasmid DNA and the number of enrichment PCR cycles used during the Illumina library preparation (Table 1). Illumina sequences from these single-plasmid controls revealed significant counts of non-canonical barcode sequences (Fig 2). The percent of errors in raw data were high and ranged from 3.71% to 6.11% across replicates (Table 2, ‘Raw’ column). Altering the number of PCR cycles or input DNA concentration had little effect on the variation we observed, thus indicating that most of the barcodes identified were due to errors in and leading up to DNA sequencing.

**Table 1:** Single-plasmid controls preparation.

Single-plasmid control	Barcode	Plasmid copies	PCR cycles
1P-A	TCACAGGGGTAA	1.00E+02	40
1P-B	TCACAGGGGTAA	1.00E+03	35
1P-C	TCACAGGGGTAA	1.00E+04	32
1P-D	TCACAGGGGTAA	1.00E+05	29

**Table 2:** Percent of erroneous barcodes in Illumina sequencing reads of single-plasmid controls: original (raw), quality thresholds for any base with each of two thresholds, and clustering with different Levenshtein distances applied.

		Quality Cutoff		Clustering		
Control	Raw	20	30	L1	L2	L3
1P-A	3.71%	2.40%	1.19%	0.51%	0.06%	0.03%
1P-B	6.11%	5.04%	4.06%	0.98%	0.03%	0.02%
1P-C	4.23%	2.76%	1.65%	0.73%	0.07%	0.04%
1P-D	5.07%	3.65%	2.55%	0.97%	0.06%	0.03%

The high error rate is striking. Inspection of Fig 2 reveals the nature of deviations from the true barcode. For a 12-mer, there are 37 possible 1-step deviations that ignore the identity of the change: 12 single-base changes, 13 single-base insertions and 12 single-base deletions. If the identity of the new base is accounted for, the 37 increases to 100 possible 1-step deviations. In these controls, we observed single-base changes and base deletions, but not all possible types were equally represented. If virus propagation was involved, this error rate could be explained as some type of virus intolerance of the insert. But this control was based on a plasmid containing a virus genome with a single barcode.

We considered the three methods of error correction for these controls. To apply the abundance threshold method for this control, we imagine that it would be known that only a single true barcode existed in the library. The abundance threshold would thus retain only the most abundant observed barcode, and it would be the true one; only a few percent of reads would be discarded and those would comprise all the errors. Thus, the abundance threshold method would perform perfectly for this case. Applying a sequence quality threshold (PHRED score of 20 or 30) for any individual base eliminates some of the error, but not all (Table 2); by itself, this method does not eliminate enough of the error to suffice. Separately of the other methods, clustering (with L = 1, = 2, = 3) shows progressive improvement in eliminating error and is superior to the quality threshold. Virtually no erroneous barcodes remain after applying L = 3.

### Multiple-plasmid controls of unequal abundance: One type of clustering is effective

A second set of controls comprised 10 plasmids containing known different barcodes at different concentrations. This type of control is appropriate whenever barcoded genomes are not equally abundant (although the abundance skew appropriate for the controls may not be clear without looking at the abundances of reads in the actual libraries). Again, we varied the number of enrichment PCR cycles used during the Illumina library preparation (Tables 3 and S1). These controls take a step toward generating a typical dataset while still allowing detailed *a priori* knowledge of the source barcodes.

**Table 3:** 10-plasmid controls preparation.

Barcodes	10P-A		10P-D		10P-G	
	Plasmid copies	PCR cycles	Plasmid copies	PCR cycles	Plasmid copies	PCR cycles
TCACAGGGGTAA	1.00E+05	29	1.00E+04	29	1.00E+05	22
ACAAGACCGGAA	1.00E+03		1.00E+04		1.00E+05	
ATATAGAGCTGT	1.00E+03		1.00E+04		1.00E+04	
ACATACCTGCTA	1.00E+02		1.00E+04		1.00E+04	
GTGTCAGGCACA	1.00E+02		1.00E+04		1.00E+03	
TGCCACTCTAGC	1.00E+01		1.00E+04		1.00E+03	
CTCGATTCACTC	1.00E+01		1.00E+04		1.00E+02	
GAACCCGTGGAA	1.00E+01		1.00E+04		1.00E+02	
CTGTATATTTTA	1.00E+01		1.00E+04		1.00E+01	
GAAACCATGACA	1.00E+01		1.00E+04		1.00E+01	

**Fig 3:**
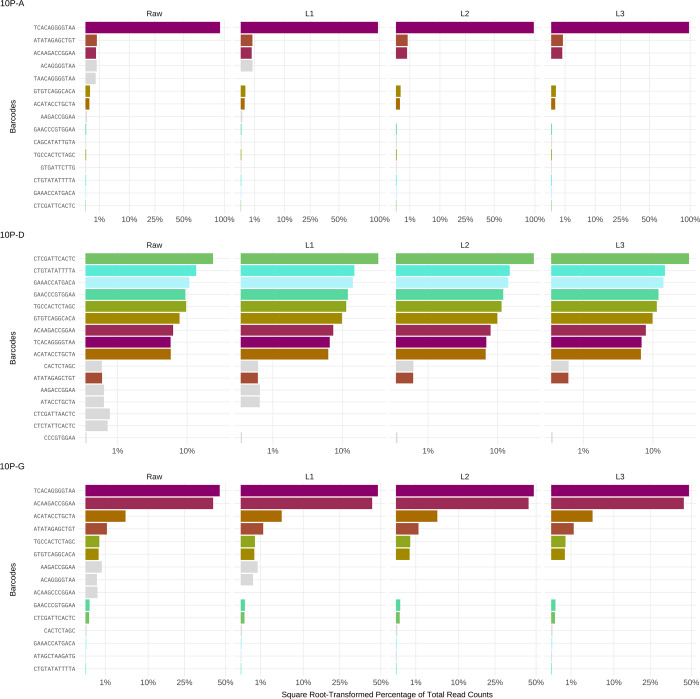
Illumina sequencing reads from 10-plasmid controls using different clustering distances. The y-axis depicts the barcode sequence; the x-axis shows the square root-transformed percentage of total read counts. The colored bars represent the control barcodes. Gray bars represent the most common erroneous barcodes within a library. The plots compare the raw percentages (no clustering) with clustering using Starcode’s message-passing algorithm, and L = 1, L = 2, and L = 3 distance parameters. Here we show the 10-plasmid controls 10P-A, 10P-D and 10P-G. Additional 10-plasmid controls are shown in S4 Fig.

As with the single plasmid controls, a substantial error rate was observed. In contrast to the 1-plasmid controls, the abundance threshold method now fails. The top 10 most-abundant barcodes include several erroneous ones, omitting the true barcodes incorporated at lower concentrations (“Raw” column in Figs 3 and S4). With base quality cutoffs discounted from the 1P controls, we are left with clustering to control the error.

Starcode offers a set of related clustering procedures. The Levenshtein distance is one parameter that may be varied, so is the basis of whether and how to cluster two different barcodes. The 10-plasmid controls allow us to resolve choices among some of these other Starcode options. One critical decision in clustering depends on asymmetries in read counts: if two barcodes differ by L = 2 (for example), should they necessarily be combined? By Starcode’s greedy “spherical” algorithm, they would be combined if they lie within the set threshold distance [17]. In contrast, by Starcode’s message-passing algorithm they would be combined only if the abundances of the two barcodes were sufficiently different; this algorithm should thus be prone to cluster erroneous (hence less-common) barcodes with true ones. The message-passing algorithm begins with the lowest-count sequences and gradually builds up clusters taking relative count numbers into account. The centroid is defined as the representative barcode (which should often be the parent). Two clusters are combined into a larger cluster only if the centroids are within the threshold distance, and the total counts of one cluster at least 5 times times larger than the counts of the other. The condition on relative count sizes addresses the likelihood that an erroneous barcode will have far fewer reads than a true barcode. If two true barcodes happen to be within the distance threshold, more likely than not, their counts do not differ by more than a factor of 5. When applied to the 10-plasmid controls, we observed that the message-passing algorithm reduces noise at lower values of L as compared to the greedy spherical algorithm (S5 Fig). Applying different Levenshtein distances using Starcode’s message-passing algorithm to the 10-plasmid control series, we observe that distances of L = 2 or L = 3 substantially reduced errors (Figs 3 and S4). However, the smallest distance among the 10P control barcodes was L = 4, and only one of the 45 possible pairs was that close. It is thus not expected that different true barcodes could have been combined with L = 3.

**Fig 4:**
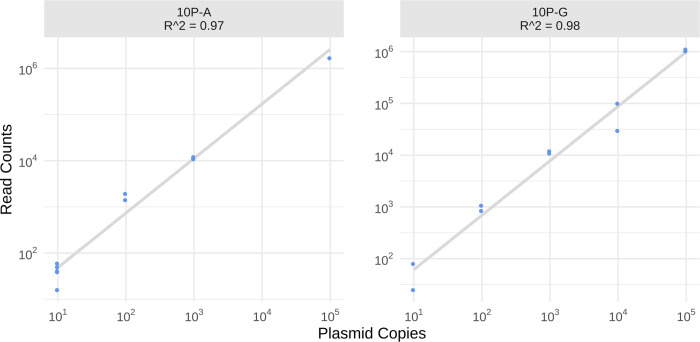
Linearity plots of 10-plasmid controls with L3 clustering parameter. The log10 transformed x-axis shows the copy number of plasmid inputs, the log10 transformed y-axis represents L3 clustered read counts. Linear regression trendlines are plotted in gray, with corresponding R^2^ values. Linearity of the 10-plasmid control series 10P-A and 10P-G is shown (the linearity of additional 10-plasmid controls is shown in S6 Fig).

Finally, we also used the 10-plasmid control to probe for linearity of recall of differentially abundant barcodes and to determine the limit of detection. Raw counts of the 10-plasmid controls show a linear recall of barcodes of at least three orders of magnitude with a sensitivity reaching as low as 10 input copies per enrichment PCR in most controls (Figs 4 and S6).

### Considerations when applying clustering

As barcode identifiers become increasingly prevalent across NGS techniques, new algorithms for correcting barcode errors have been developed. In addition to Starcode, other popular software includes Calib [18] and Bartender [19]. Calib is intended for paired-end biological sequence data sandwiched between UMIs, and uses both UMI and sequence similarity for clustering. Our data is single-ended, and is exclusively the barcode identifier itself, which is not what Calib is designed for. Bartender would apply to our data. Bartender and Starcode have fundamental similarities. Both use count data for every individual barcode, in addition to the sequence itself, as crucial information in clustering. Both Bartender and Starcode perform clustering of hundreds of thousands or millions of reads in a short amount of time. We prefer Starcode in part because of the simplicity of its algorithm, and in part because it uses Levenshtein rather than Hamming distance as a measure of sequence similarity. While most of our barcodes have the intended length of 12 nucleotides, our data do contain barcodes of varying length, which suggests a distance metric that takes indels into account. However, we make no claim about the superiority of Starcode over Bartender, or the other way around. Indeed, the superiority (if any) of one method over another may well vary with the application and the nature of errors.

How does one decide which Starcode parameters to apply? Clustering has the benefit of eliminating error (by associating erroneous barcodes with the true parent) but risks combining different parents as well as combining erroneous barcodes with the wrong parent. Combining different parents is the lesser problem because combining different parents merely reduces the effective number of barcodes, it does not lead to mis-assignments of barcode parent.

The challenges of clustering depend on the design of the library plus the magnitude and types of error. If nearly all parent barcodes differ by more than twice the distance created by errors, then there can be few mistaken clusterings when the error is known; sparser libraries are better, but the gain with sparseness will rapidly attenuate. Our library was approximately 5,000 randomized 12-mers. Although there are almost 17 million possible 12-mer barcodes, such that 5,000 fills a small fraction of this space, the expected distances give a somewhat different picture: approximately 98% of 5,000 random barcodes are at least a distance of L = 1 from each other, 75% a distance of at least L = 2, but only 2–3% are at distance L = 3 or more. With these distances, it would seem that clustering out to L = 3 should then make many mistakes. This, however, is where the asymmetry of Starcode message-passing has an important role: two barcodes will be combined only if one is 5x greater than the other. Thus, two parent barcodes will be clustered together only if they are within L = 3 AND one is many times more common that the other.

What about combining progeny barcodes that have errors with the wrong parent? As noted above, there are 100 different single-step mutations possible from a barcode. The vast majority of these changes are therefore unlikely to render the progeny of one barcode closer to another barcode within L = 3.

To test the error in our clustering method for 5,000 barcodes, we ran a simulation (described in Methods). Unlike the one- or ten-plasmid controls, we have no *a priori* knowledge of the true barcodes in the plasmid library. For our simulation we generated a set of 5,000 random 12mers, and mimicked the range of counts and errors we found in the control and viral libraries. After our procedure of clustering using Starcode with L = 3, and a 99% read cutoff, we found 82% of the original random 12mers were recovered. There were no centroids (after clustering and cutoff) that weren’t among the original random 12mers.

In light of these considerations and the analyses of these controls, we conclude: (i) Applying the message-passaging algorithm and a clustering distance of L = 2 or 3 should consolidate most of the variation with the true barcodes. (ii) Differences in PCR cycle amplification did little to affect the relative abundance of barcoded virus genomes. (iii) At least on a small number of variant barcodes with well-defined inputs, our approach provides a linear recall spanning a range of at least three orders of magnitude.

### Analysis of the plasmid library

One of the steps for the preparation of a barcoded muPyV stock requires the generation of a pool of plasmids containing the barcoded virus genome (similar to plasmids in our known barcode controls above) that will be subsequently isolated from the vector, circularized and transfected into cells. As described above, we estimated the pool of barcoded plasmids to contain no more than 5,700 different true barcodes based on the estimated bacterial colonies number that make up the library. This is an unreasonable number to sequence by the Sanger method. However, understanding the barcode diversity in the plasmid library is an important prerequisite to understanding the diversity of our virus library. We therefore applied Illumina sequencing to the plasmid library.

**Fig 5:**
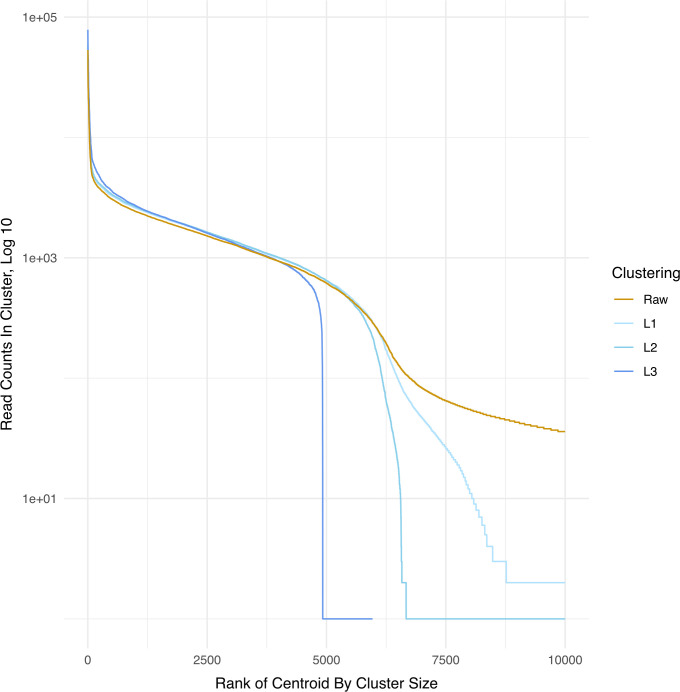
Comparison of barcode counts between raw and different clustering distances. This figure shows that the number of barcodes that are called decreases with the increasing clustering distance; clustering with L = 3 substantially decreases the barcode counts called in the raw sequencing reads. Note that the highest-count cluster is ranked 1. The figure is cut off to include only the 10,000 most abundant barcodes to focus on the “elbow” where the number of barcodes that are called display a steep drop-off.

We compared the distribution of barcode ranks in the plasmid library (based on read abundance) between the raw reads and clustered reads using L = 1, 2, and 3 (Fig 5). The curve of raw (unclustered) barcodes ordered by rank exhibits a shallow shoulder centered near the 6,000th barcode but extending to the 200,000th (note, the cutoff for the graph in Fig 5 is at 10,000 barcodes). This shoulder indicates a marked drop in abundance of reads per barcode, consistent with the less abundant barcodes resulting from error (note, the log scale used greatly inflates the apparent abundance of less common barcodes). As clustering progresses from L = 1 to 2 to 3, a pronounced shoulder materializes just under 5,000 barcodes, a number broadly compatible with the estimated size of the plasmid library.

After clustering with L = 3, the fewest reads of any barcode numbered several hundred, and ~ 3,800 barcodes had at least 1,000 reads (Fig 5). However, there is considerable variation in the number of reads across clustered barcodes, with approximately 100-fold more reads for rank 1 than for rank ~ 4,000. The distribution of read numbers for clustered barcodes broadly matches that for unclustered barcodes up to rank of 4,000 (Fig 5), demonstrating this large variation is not an artifact of clustering.

These results show that true barcodes in the plasmid pool are not evenly distributed across the pool. Importantly, there is a ~100-fold difference in abundance between the most common and least common cluster for the top 4,000 most abundant barcodes, and this difference rises to 10,000-fold when including the remaining lower abundance barcodes (~5,000 total). For our 10P plasmid controls, where we deliberately created differences in DNA abundance, those differences were faithfully reproduced in the Illumina sequencing reads (Fig 4). This suggests that these apparent large differences in our plasmid library are not introduced by sequencing artifacts. This apparent large variability in plasmid counts may well arise from differences in amplification and/or propagation of the plasmids.

This abundance skew has several consequences. (i) It may explain the observation of only 20 unique barcode sequences in the sample of 21 bacterial colonies cloned from virus barcodes (described above). (ii) It shows that the abundance of plasmids used in our 10P controls are approximately representative of the library. (iii) It exposes a problem with the ‘cutoff’ error correction method that (in our case) would exclude all but the ~5,000 most-abundant barcodes: for example, erroneous reads from the most abundant barcode may be more abundant than true reads from lower abundance barcodes. These data therefore reinforce the use of clustering for error correction instead of merely eliminating barcodes based on abundance.

To further check the difference between the ‘top 5k’ cutoff approach and the message-passing L3 clustering, we assessed the intersection of the two approaches. There was 91% agreement (4,553 barcodes in common), suggesting considerable agreement and that use of a cutoff might be acceptable for some purposes (however, this agreement declines when using the virus library–see below). Additionally, these data equally suggest that clustering is recovering true barcodes.

**Fig 6:**
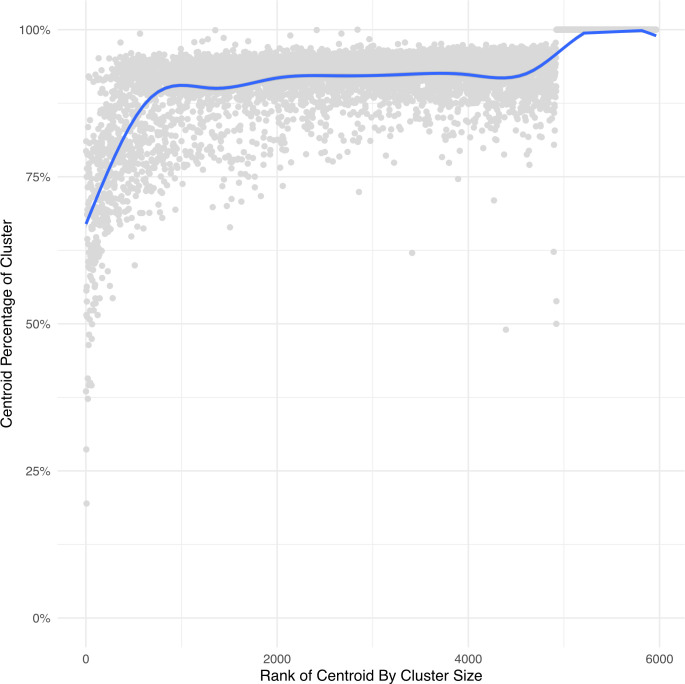
Centroid counts as percentage of cluster counts. Each gray dot represents the percentage of counts in a cluster (using L3 clustering) from the plasmid library that comes from its centroid. The centroids are ordered by the size of their cluster. The highest-count cluster is ranked 1. A smoothed curve (in blue) is fitted to the dot plot. This plot shows that the majority of cluster counts come from the defined centroid.

Every cluster consists of several barcodes, one of which serves as the representative for the cluster (also known in as the “centroid” in the literature). To get a sense of how much the clustering contributes to the total abundance of the barcode compared to the centroid, Fig 6 shows the proportional contribution of the centroid to the cluster. The clusters represented on the right consist of a single barcode of count 1, thus the “centroid”(the barcode itself) trivially contributes 100% of the reads to the cluster. For the remainder, we see that aside from some rare exceptions, centroids contribute the overwhelming majority of counts to their clusters. This is consistent with the expectation that clusters represent error-correction of what are likely the "true" barcodes, which are assumed to be the centroids.

### Using a cutoff to avoid severe barcode deviations in the plasmid library

**Fig 7:**
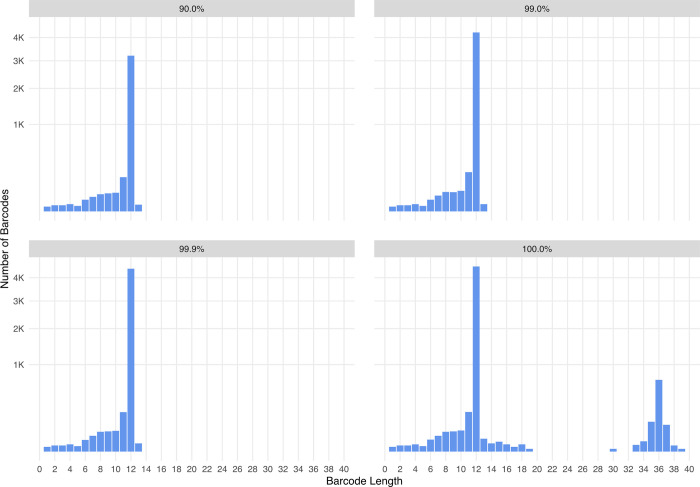
Barcode length distributions for 90%, 99%, and 99.9% sequencing reads cutoffs. Applying a cutoff for barcode abundance eliminates most recall of longer barcodes that were not intended in the original design for a 12-nucleotide barcode library. These barcodes are derived from the L3 clustering. The y-axis is square root-transformed so low values are more visible.

As expected from the design of the library, the majority of barcode lengths recalled were 12 nt long (Fig 7). However, a small subfraction of barcode reads had a median length of 36 nt, even after clustering. These were clearly not intended in the experimental design for construction of the library and were of very low abundance. Given their existence in the library, we reasoned there must be some aberrant, low-count barcodes that remained in the library even after clustering, whether through the barcode extraction step, or some upstream anomaly in the library preparation. Using a length criterion to remove long barcodes would solve the long-barcode problem, but given the possibility of other anomalies, we chose to remove low-count barcodes instead. We therefore applied a series of cumulative read count cutoffs (top 90%, top 99%, or top 99.9% most abundant, as described in Fig 1) to weed out these low abundance likely artifacts. (A cutoff of 90% means that the remaining barcodes account for 90% of the total counts.) This analysis showed that all three cutoffs removed most of the aberrant size barcodes. The difference in the number of barcodes left after applying a 99% or 99.9% cutoff was small, and we therefore applied a conservative cutoff of the top 99% most abundant reads to subsequent work on the virus library. After applying the cutoff (following clustering), barcodes of length 12 nt account for 88% of the total count. Barcodes of length 11 or 12 nt account for 92% of the total count.

**Fig 8:**
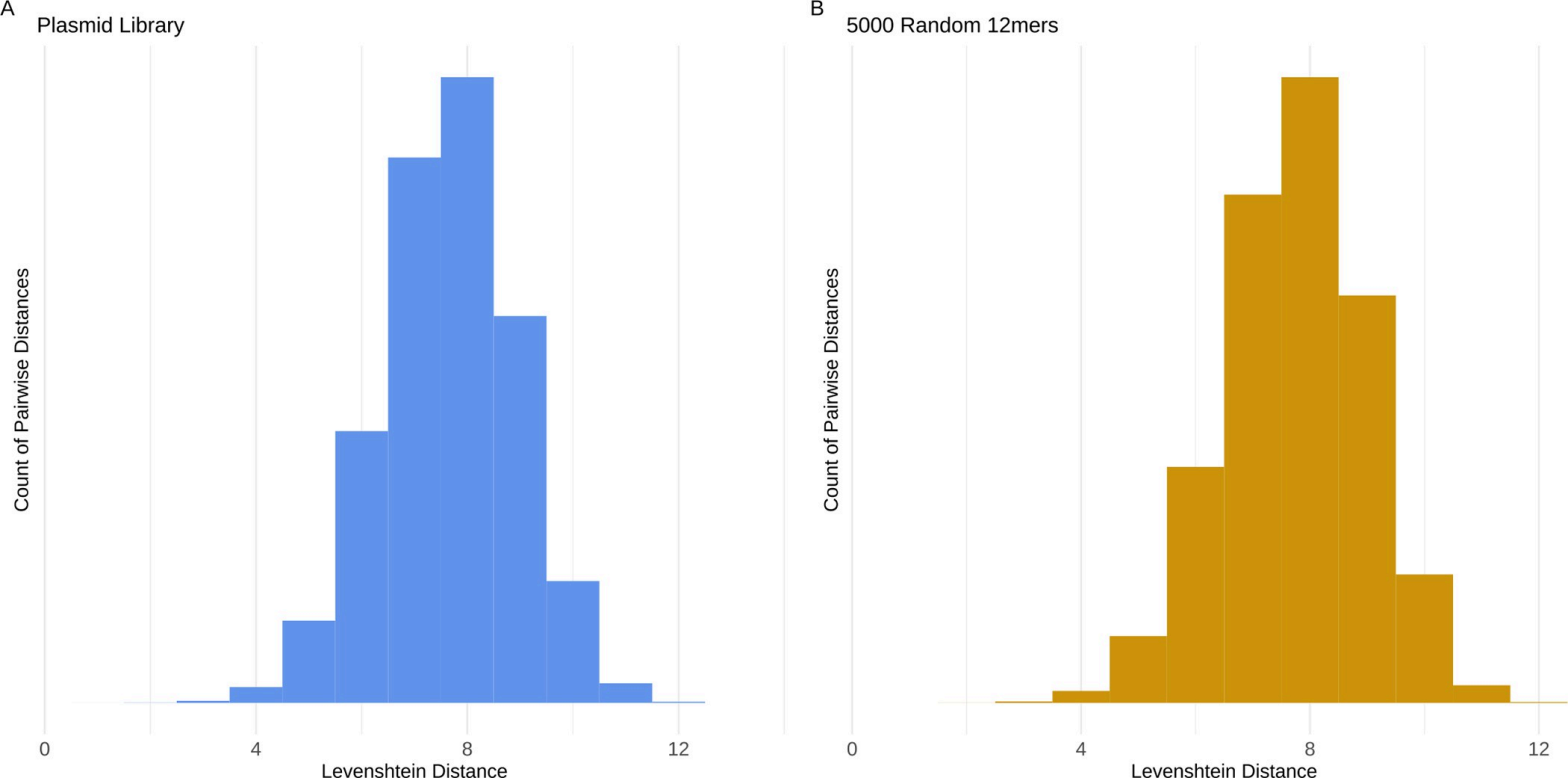
Distributions of the barcode pairwise distances within the plasmid library (panel A, shown in blue) and 5,000 randomly generated 12mers (panel B, shown in yellow). The figure represents the pairwise Levenshtein distances between centroids in the plasmid library after applying a L3 clustering distance and a 99% reads cutoff. The 5,000 randomly generated 12mers are not clustered, and with no cutoff. Data show that the proportion of possible barcode sequence space covered is similarly sparse in both cases.

We evaluated barcode properties of our plasmid library after clustering and applying the 99% cutoff (Fig 8A). The histogram of pairwise Levenshtein distances between barcodes shows a narrow peak at 8. This indicates that the barcodes are well-separated, which will help to properly assign barcodes in downstream experiments that may have a small number of errors associated with a true, parent barcode. As a comparison, we generated a random set of 5,000 12mers and calculated the pairwise distances between those (Fig 8B, see Methods for more details on simulation). The distribution of pairwise distances and size distribution were consistent with a randomly-generated barcode library that arose as expected from the experimental design.

In summary, our analysis of the plasmid library shows (i) there is more variation in barcode abundance than intended in the original library design, (ii) that clustering works in eliminating the tail of low abundance (and most likely error) reads, (iii) applying a 99%-most-abundant-reads cutoff eliminates most unintended aberrant sized inserts, and (iv) centroids are usually the dominant member of a cluster consistent with the clustering approach working as intended in identifying true barcode sequences.

### Clustering of barcodes in the virus library

**Fig 9:**
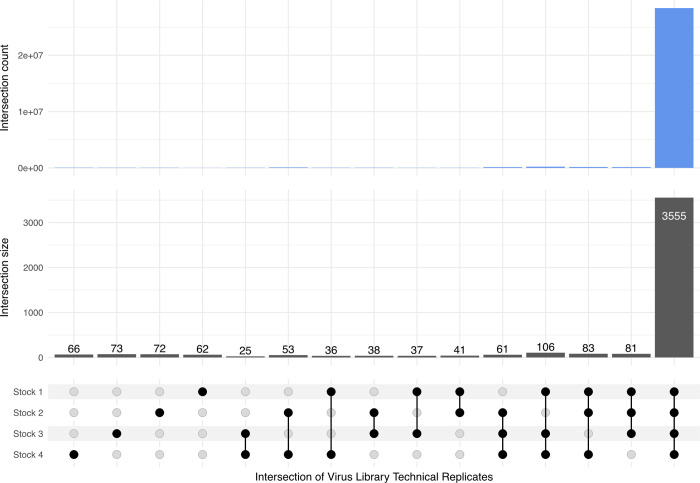
Technical replicates of the sequencing of the virus library. Using Illumina sequencing, one replicate of the virus library was sequenced in one run and three additional technical replicates were sequenced in a separate run. Thus, the four technical replicates were sequenced in two independent runs. The UpSet plot shows the number of barcodes that intersect amongst the four virus library technical replicates (lower panel), as well as the total counts of those barcodes in blue (upper panel). The libraries were clustered using L3 distance and a 99% reads cutoff was applied. The four replicates show a large degree of overlap in clustered barcodes.

We created the barcoded virus stock by transfecting cells with virus genomes that had been isolated from the plasmid library and circularized. To estimate the barcode composition of this library, we sequenced this library four times in two separate sequencing runs (one technical replicate of the virus library in the first sequencing run and three technical replicates in a second run) and for each we applied the message-passing algorithm with clustering Levenshtein distance of 3 with a 99% cutoff. We compared the overlap between the technical replicates and determined that most barcodes were recalled in all four replicates (Fig 9). The sizes of the 4 technical replicate libraries after clustering and cutoff were remarkably similar, varying from 3,985 to 4,001 barcodes. Comparison of the four clustered virus libraries shows 3,555 barcodes shared across all 4 libraries, with another 331 shared across 3 libraries. This overlap is stark, with nearly all counts occurring in intersection of all 4 replicates. Using this method provides an estimate of approximately 3,600 high confidence barcodes.

**Fig 10:**
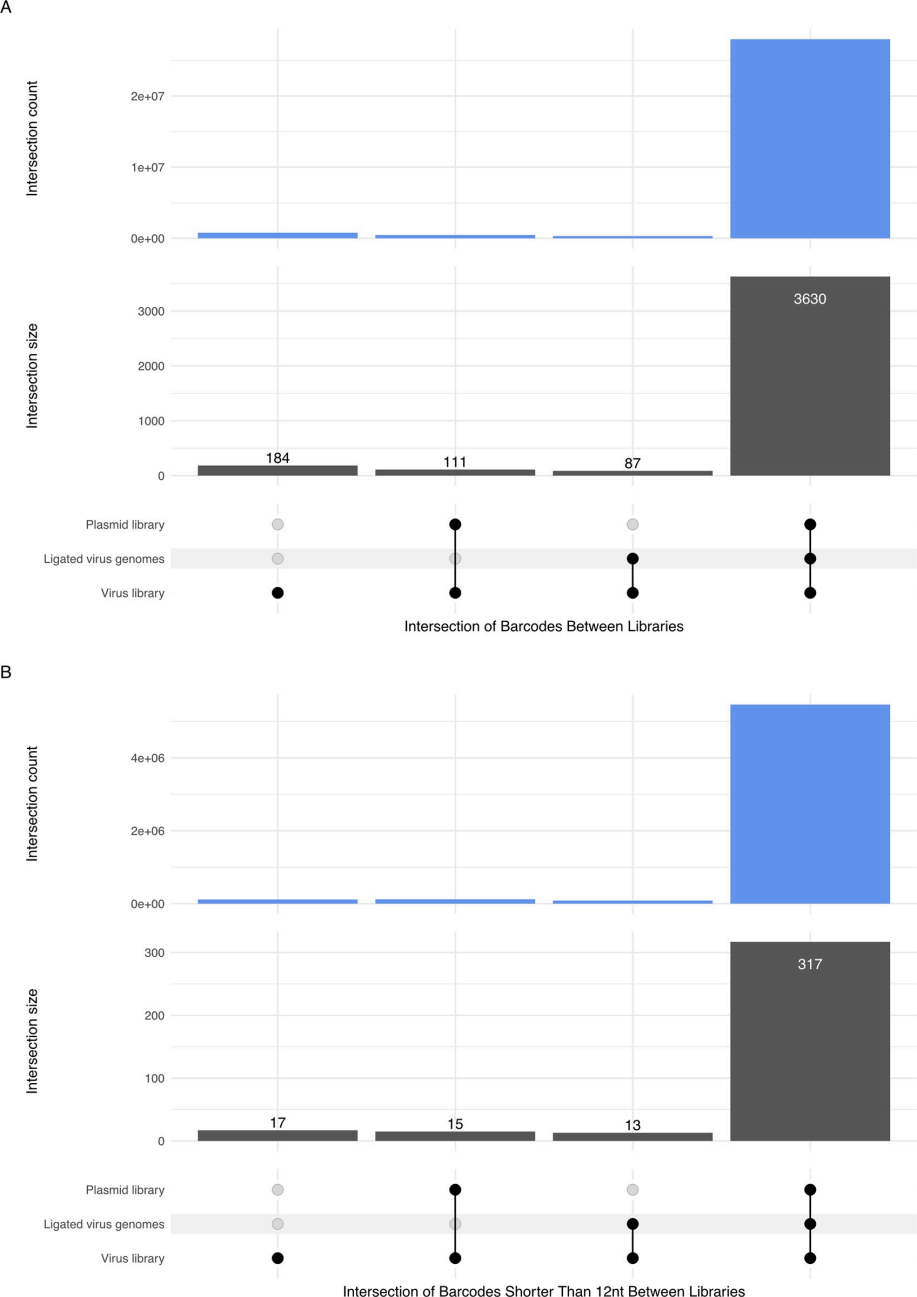
Overlap of clustered barcodes from the plasmid library, ligated virus genomes, and the virus library. A. L3 clustering distance of barcodes along with a 99% cumulative count cutoff was applied to the three libraries. In the lower panel, the UpSet plot shows the number of barcodes from the virus library that intersect with barcodes from the plasmid library and/or the ligated virus genomes. Data show that the vast majority of barcodes from the virus library are present in the plasmid library and the ligated virus genomes. In the top panel, the UpSet plot represents the total read counts associated with barcodes in the virus library that intersect with the plasmid library and the ligated virus genomes. Data show that the barcodes in all three libraries account for the overwhelming number of counts in the virus library. B. Overlap of clustered barcodes that are shorter than 12 nucleotides from the plasmid library, ligated virus genomes, and the virus library. L3 clustering distance along with a 99% cumulative count cutoff was applied to the three libraries. In the lower panel, the UpSet plot shows the number of barcodes from the virus library that intersect with barcodes from the plasmid library and/or the ligated virus genomes. Data show that the majority of short (<12nt) barcodes in the virus library are also present in the plasmid library and ligated virus genomes. In the top panel, the UpSet plot represents the total read counts associated with shorter barcodes in the virus library that intersect with the plasmid library and the ligated virus genomes. Data show that the shorter barcodes that overlap in all three libraries account for the majority of shorter barcodes in the virus library, suggesting that they did not arise during the course of infection.

To determine potential bottlenecks in the process of generating the barcoded virus library, we compared the overlap between the virus, plasmid, and control ligation libraries that were used to give rise to the virus library. After clustering and cutoff, the majority (90%) of the virus barcodes from the pool of our 4 replicates of the virus library were recalled in both our sequencing of the plasmid libraries and the control ligated virus genomes, with 5% of the virus barcodes being unique to the virus library. Examining the counts, 95% of the counts for the pool of the virus library are for barcodes that are also found in both the plasmid library and ligated virus genomes, and only 3% of the counts were unique to the virus library barcodes (Fig 10A). We note that these numbers compare centroids of clusters; the similarity of the libraries may in fact be even higher, as the barcode chosen as the centroid of a cluster may differ between libraries, even if the clusters themselves are very similar. We conclude that the vast majority of barcodes present in the plasmid library are incorporated into the virus library. Thus, as would be predicted, a greater repertoire of barcodes could be obtained in the virus library if plasmid libraries are derived from an increased number of unique bacterial colonies.

Overlap between the top 5k approach and message-passing L3 clustering was 80% (down from 91% for the plasmid library). This is a substantial drop and reinforces the utility of clustering. In addition, there was only 35% overlap between the top 5k uncorrected barcodes and sphere-based L3 clustering for the viral library. This latter comparison reinforces the benefit of using message-passing clustering over the sphere-based clustering. To the extent that the top 5k barcodes contain a significant number that are within L = 3 of each other, the sphere-based algorithm will assimilate those barcodes into single clusters.

After clustering using Starcode with L = 3, and applying a 99% cutoff, most barcodes were the expected size. We noted that approximately 9% of barcodes were less than 12 nts (ranging from 0–11 nucleotides in length). We determined that 88% of these shorter barcodes were also present in the plasmid library and ligated virus genomes that gave rise to the virus stock (Fig 10B). Combined, these findings do not support overt pressure for viruses to lose the barcode under our experimental conditions, although we note that one of the PCR amplification primers overlaps a portion of the 3’ end of the synthetic insert so we cannot rule out some viruses having lost the entire insert. Overall, these results suggest that while some shorter barcodes may arise during virus propagation, shorter-than-expected barcodes mainly arise due to synthesis errors in the primers used to generate the barcoded plasmid libraries and/or deletions that occurred during production/propagation of the plasmids.

**Fig 11:**
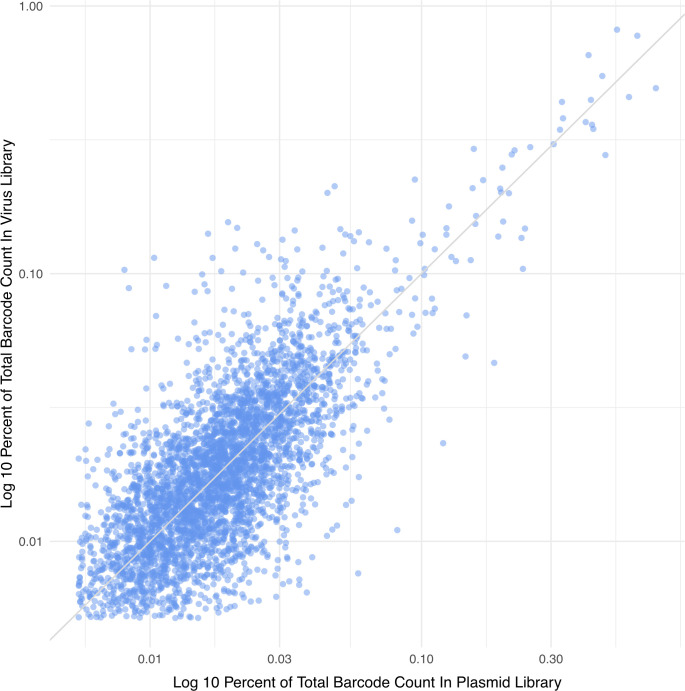
Abundance of clustered barcodes in the plasmid and virus libraries. For each barcode, its log10 percentage of counts in the plasmid library is plotted on the x-axis, and its log10 percentage of counts in the virus library is plotted on the y-axis. A trendline with slope 1 through the origin is plotted. Data show a correlation with the more abundant barcodes in the plasmid library generally giving rise to the more abundant barcodes in the virus stock.

Given that the plasmid library had large differences in the relative abundance of its members, we asked if the virus library barcode abundance was reflective of this. We observe differences in individual barcode abundance in the virus library (L3 clustered, 99% cumulative count cutoff) ranging between 1,544 counts for the least abundant up to 243,503 counts for the most abundant barcode. There is good correlation between the abundance of plasmid and virus library barcodes, both after clustering and cutoff, with a Pearson correlation of 0.87 (Fig 11). Combining the above findings, these results suggest that, after the original transformation of bacteria, there were no major bottlenecks in generating virus, with the virus library largely reflective of the composition of the plasmid library used to give rise to it.

In summary, (i) clustering under our conditions of the virus stock results in highly similar clusters between technical replicates, supporting the good reproducibility of our approach, (ii) there is a large agreement of clusters between the virus and plasmid libraries both in identity and abundance, and (iii) under the conditions we applied, after generation of the plasmids that give rise to the virus library, the virus library was not subject to major bottlenecks during its generation. We conclude that the wet bench and computational approaches developed here are suitable for tracking longitudinal parallel infections with barcoded microbes.

## Discussion

Conducting parallel infections with multiple variants of a pathogen is a longstanding way to assay competitive fitness advantages as well as to track the dynamics of infection [8,9,20–26]. Studies that employ genetically tagged viruses can track the comparative progression of infection based on differences in abundance of each variant. In its simplest form, this approach can be applied to as few as two differentially marked pathogens [25–29]. The availability of large scale massively parallel sequencing greatly expands the number of differentially marked pathogens that can be assayed in a single experiment. Indeed, recent studies using RNA viruses or retroviruses have employed this technique to study a variety of aspects of infection [3,5,6,30–33]. However, basic questions about how best to employ this approach, such as library generation strategy and the optimal computational methods to interpret sequencing data remain poorly characterized.

Here we described the construction of a barcoded muPyV (murine polyomavirus) library. We knew that the library could be comprised of no more than 5,700 different source barcodes, yet sequencing of the final library retrieved close to half a million apparent barcodes, indicating a huge level of error. This error was possibly due to Illumina sequencing of low complexity of amplicons [16] (despite our use of offset staggered multiplexing primers and spiked PhiX DNA). The error rate of raw reads was so severe that attempts at uncorrected barcode identities would have led to unacceptable errors in barcode interpretation.

Thus, a major goal of this study was to solve this barcode interpretation problem. We developed and tested several error-correction approaches based on empirical controls and computational analyses. We expect our analyses to be helpful in future studies of barcoded microbes, as it seems likely that a high barcode error rate will plague other studies as well.

The main resolution of our study is to propose a top-down protocol for future studies using barcodes:

1. Design the barcode library to be appropriately sparse in the sequence space of possibilities. The appropriate sparseness will depend on error rates. As a rule, when designing random libraries, allow for at least millions of possible barcodes for every barcode implemented. Generally, when a microorganism can tolerate a larger barcode without fitness penalty, larger barcodes are desirable.
2. Analyze control barcode sequences to measure the magnitudes and types of error. This information may then be used to back-calculate library sparseness.
3. When analyzing the completed library, computationally resolve barcode errors with a method tailored to the observed problems. If the library is appropriately sparse, a variety of methods may be suitable. When errors comprise a major problem, methodology will present a challenge. Multiple methods may need to be combined.

The methods of error correction tested here included (i) eliminating barcodes represented by ‘few’ reads, (ii) eliminating barcodes with low-quality bases, (iii) clustering barcodes based on sequence similarity and dissimilarity in abundance. From our controls, we found the first two approaches to be inadequate. Clustering offered the most satisfactory approach.

However, not all clustering methods proved equal. The method we adopted here was a ‘message-passing’ algorithm in which clustering began with low copy barcodes and progressed toward the more abundant barcodes. Furthermore, two barcodes were combined only if their abundances were appropriately asymmetric: one was at least 5x as abundant as the other. We suspect that this asymmetry is especially suited to error correction whereby the erroneous barcodes are much fewer in number than their non-mutant parents.

In contrast, many common clustering algorithms for sequences use top-down “greedy” algorithms, where the highest count or longest sequence is considered first, and all sequences within some threshold distance are made a cluster with the initial sequence as the representative, or “centroid”. The popular CD-HIT clustering software [34] works in such a way: the longest sequence is considered first, with all sequences within a threshold distance clustered with that sequence. Then the next-longest unclustered sequence is considered, and the process repeats. In applications where sequence lengths differ considerably, and longer sequences are generally considered higher-quality, this is a reasonable approach. In the case of barcodes, the situation is different. Our barcoding approach aimed to achieve consistent sequence lengths, in this case 12-mers. Rather than large variation in length of sequence, we observed large variation in counts, spanning orders of magnitude. For this situation, which we expect would be encountered by others wishing to utilize nucleic acid barcoding, we demonstrated that using the Starcode message-passing clustering algorithm with Levenshtein distance of L = 3 (or 2) is appropriate.

Controls were essential in resolving several problems. Foremost, controls with a single parent barcode unambiguously revealed the magnitudes of errors. By examining different ratios of absolute amounts of plasmids containing known barcoded genomes, we were able to optimize clustering parameters to ensure accurate recall without loss of barcode resolution. By applying different PCR amplification cycles we also determined a range of PCR cycle conditions that did not result in substantial recall of incorrect barcodes. These conditions support accurate recall spanning at least three orders of magnitude of different amounts of barcode and sensitive to approximately 10 copies of barcode per reaction. Combined, using a prototypic model virus, we have developed a molecular and computational methodological approach we expect applicable to more broadly studying the dynamics of pathogen infection.

The ratio of the most abundant to least abundant barcode in the clustered virus library with cutoff was about 150-fold. This likely occurred because the barcode representation in our plasmid library that gave rise to the virus library spanned a similar range. This fortuitously allowed us to conclude that the ratio of virus barcodes tracks almost linearly to the barcodes in the input plasmids, which might be useful for those wishing to purposely generate libraries with different barcodes spanning a range of input copy numbers.

Our study went only so far as to resolve barcode errors in libraries (for which we relied heavily on clustering). Further considerations will apply to the analysis of downstream uses of those libraries. For example, the dynamics of a virus infection may dramatically impact the barcode counts, and the centroids of some clusters, especially low abundance true barcodes, may not be consistent between samples. Therefore, our results suggest an approach where first the “true” barcodes are identified in the initial virus library (as described in this work). Then, counts from infected experimental samples should be associated with these “true” barcodes. This could be accomplished by grouping with the nearest true barcode identified in the virus stock, where nearest is determined by Levenshtein distance, or more simply by counting only experimental sample barcodes that share sequence identity with true barcodes identified in the original virus stock.

In conclusion, we generated a library of a barcoded DNA virus. We describe unexpected outcomes of different steps of the construction of this library, devised control studies to explore optimal conditions for interpreting Illumina sequencing of barcoded pathogen libraries, and provide a validated wet bench and computational approach that may be useful to others wishing to employ barcoded microorganisms.

## Material and methods

### Cells and viruses

#### Cells

The NMuMG cells (ATCC, # CRL-1636) were kindly provided by Prof. Aron Lukacher and maintained in DMEM (Corning, # 10-013-CV) supplemented with 10% (v/v) fetal bovine serum (Corning, # 35-015-CV) and 1% (v/v) penicillin-streptomycin (Corning, # 30-002-CI) [12].

### Viruses

#### muPyV PTA wild-type stock generation

pBluescript-sk+ vector containing the full genome of the muPyV PTA strain (GenBank, accession No U27812) was digested by BamHI-HF (New England Biolabs, # R3136L) to separate the muPyV genome from the vector and gel purified. Then, the muPyV genome was self-ligated overnight at 16°C using the T4 DNA ligase (New England Biolabs, # M0202L) and purified prior transfection into NMuMG cells using the Lipofectamine 2000 reagent (Invitrogen, # 11668019). NMuMG cells were cultured until 90% cytopathic effect (CPE) was reached (5 days post-transfection). The cells and the supernatant were collected, subjected to freeze/thaw cycles and cleared by centrifugation at 4°C. Fresh NMuMG cells were infected with the crude lysate at 37°C and cultured until 90% CPE (day 6 post-infection). The infected cells and the supernatant were harvested, subjected to freeze/thaw cycles, buffered at pH 8.5, incubated at 42°C for 30 min, vortexed to release muPyV and cleared by centrifugation. The muPyV PTA stock was aliquoted and stored at -80°C until use.

#### Construction of the barcoded muPyVs library

pBluescript-sk+ vector containing the full genome of muPyV PTA (GenBank, accession No U27812) was amplified by reverse PCR using the Phusion High-Fidelity DNA Polymerase (New England Biolabs, # M0530L) and the 5’ phosphorylated primers BCFSB7 5’- NNNNNNCAATTGAATAAACTGTGTATTCAGCTATATTC-3’ and BCRSB7 5’- NNNNNNGAATAAACATTAATTTCCAGGAAATAC-3’ (Integrated DNA Technologies). The PCR product was digested by DpnI (New England Biolabs, # R0176L), gel purified and self-ligated using the T4 DNA ligase (New England Biolabs, # M0202L) before transforming MAX Efficiency DH5α competent cells (ThermoFisher, # 18258012). Transformed bacteria were cultured overnight in LB broth containing 100μg/mL of ampicillin and the plasmid library was purified. The number of transformed bacteria was estimated in parallel on an aliquot of the transformed bacteria by counting the colonies on LB agar plates containing ampicillin. The presence of the barcode was confirmed in plasmids from colonies by enzymatic digestion using BamHI-HF and MfeI-HF (New England Biolabs, # R3136L and # R3589L, respectively) and by Sanger sequencing using the primer FullSeq3 5’-GTTAGAGTGTATGATGGGACTG-3’. The virus library was then prepared by transfecting the plasmid library as described above for the generation of the muPyV wild-type stock. To confirm the presence of the barcode in the virus library, virus DNA was purified using the QIAamp DNA Mini Kit (Qiagen, # 51304) and the region surrounding the poly A signals was PCR amplified using Taq DNA polymerase (New England Biolabs, # M0267L) and the primers NGS_Fwd 5′-CATGGCCTCCCTCATAAGTT-3′ and NGS_Rev 5′-GAATATAGCTGAATACACAGTTTATTC-3′ following the manufacturer’s recommendation. The PCR product was purified, cloned using the TOPO TA Cloning kit for Sequencing (ThermoFisher, # 450071) and Sanger sequenced (S1 and S2 Figs).

#### muPyV titration by immunofluorescence assay

NMuMG cells were infected in duplicate with serial dilutions of the virus sample for 1 hour at 37°C. 30–40 hours p.i., cells were gently washed once and fixed/permeabilized with PBS containing 4% paraformaldehyde. Cells were washed three times, blocked with PBS containing 1% goat serum overnight at 4°C and incubated for 1 hour at room temperature with a rabbit anti-PyV VP1 antibody (a gift from Richard Consigi) diluted 1:50 in PBS. After three washes, the cells were stained with Alexa Fluor 488 goat anti-rabbit IgG (ThermoFisher, # A-11008). The number of stained cells per field was counted under an inverted fluorescence microscope (Leica), and infectious titers (IU/ml) were calculated.

### Illumina NextSeq library preparation

#### Enrichment PCR

This first step PCR amplifies a 360 bp fragment encompassing the barcoded region of muPyV genome using primers NGS_Fwd 5′-CATGGCCTCCCTCATAAGTT-3′ and ReVOUT1 5’-CAGGGTCTTGTGAAGGAGGT-3’ (primers binding site shown in S3 Fig). Briefly, the enrichment PCR reaction contained 4.77 x 10^5^ copies of muPyV genome (estimated by qPCR), 0.5μM of the above primers, 200μM of each dNTP, 1U of Phusion High-Fidelity DNA Polymerase (New England Biolabs, # M0530L) in 1X of Phusion HF reaction buffer. After an initial denaturation step for 1 min at 98°C, the amplification was performed by 22 cycles of 10 sec at 98°C (ramp 2°C/sec), 15 sec at 55°C (ramp 2°C/sec), 30 sec at 72°C (ramp 2°C/sec) followed by a final extension step for 5 min at 72°C. The DNA polymerase was inactivated at -20°C. The presence of a specific amplification product was checked on a 2% agarose gel.

#### Indexing PCR

This second step PCR amplifies a 75 bp fragment encompassing the barcode region from the enrichment PCR product, adding Illumina adapters and indexes to permit multiplexing, as well as spacers to increase the library complexity. The amplicons size generated ranges from 211 to 216bp. The sequence of the staggered indexing primers (Integrated DNA Technologies) is presented in S2 Table (primers binding site shown in S3 Fig). Briefly, enrichment PCR products were digested with 20U of ExoI (New England Biolabs, M0293L) at 37°C for 30 minutes to remove residual primers and heat inactivated at 80°C for 20 min before proceeding to the indexing PCR. Then, two indexing PCR reactions were made for each sample containing 1μl of the enrichment PCR products, 0.3μM of indexing primers, 200μM of each dNTP, 1U of Phusion High-Fidelity DNA Polymerase (New England Biolabs, # M0530L) in 1X of Phusion HF reaction buffer. After an initial denaturation step for 1 min at 98°C, the amplification was performed by 22 cycles of 10 sec at 98°C (ramp 2°C/sec), 15 sec at 54°C (ramp 2°C/sec), 30 sec at 72°C (ramp 2°C/sec) followed by a final extension step for 5 min at 72°C. The DNA polymerase was inactivated at -20°C. The presence of a specific amplification product was checked on a 2% agarose gel.

#### Amplicons pooling and quality control

The two indexing PCR reactions were merged together and concentrated using the Monarch PCR & DNA cleanup kit (New England Biolabs, # T1030L) before gel purification using the Monarch DNA gel extraction kit (New England Biolabs, # T1020L). The DNA concentration of each sample was determined using the QUBIT dsDNA BR assay kit (ThermoFisher, # Q32850). The samples were normalized and pooled together to constitute the final Illumina library. The final library was checked on a 2% agarose gel and sent to the Genomic Sequencing and Analysis Facility of the University of Texas at Austin for a Bioanalyzer (Agilent) quality control prior to the Illumina NextSeq SR75 sequencing run. To increase diversity, PhiX DNA was also included (~5% to the first run and ~34% to the second run).

#### Extracting barcodes

Barcodes were extracted from FASTQ files off the sequencer using Cutadapt [35], with the default error allowance of 10% for the linked adapters (requiring both adapters to flank the barcode) (Fig 1).

#### Clustering barcodes

Unless otherwise noted, barcodes were clustered using Starcode [17] for message-passing clustering, using a Levenshtein distance of 3. The highest-count barcode in a cluster (the centroid) was used as the representative for the cluster (Fig 1).

#### Checking for theoretical overclustering via simulation

To check the possibility that choosing a Levenshtein distance of 3 could lead to overclustering, we simulated 5,000 random 12mers. To check the distribution of the 12mers, we calculated pairwise Levenshtein distances between them using the base R adist function. To simulate the clustering itself, we assigned counts to the 5,000 random 12mers by simply copying the counts of the top 5,000 barcodes in the actual viral library, and assigning them to the random 5,000 12mers we created. We then created error barcodes based on our randomly-generated 12mers by progressively creating barcodes with random mutations to produce errors of Levenshtein distance 1, 2, … up to 10, for every original randomly-generated 12mer. The number of error barcodes of a particular Levenshtein distance for each of the random 5,000 12mers was determined by the assigned count for the 12mer, multiplied by the error rate for that distance, where the error rate was taken from the 1P-B single-plasmid control. The 1P-B control was chosen as a conservative reference, as it has the highest error rate among the single-plasmid controls. This process resulted in a library that contained the 5,000 randomly-generated (but known) 12mers with the same counts as the top 5,000 barcodes in the viral library, together with error barcodes generated using the same proportion of errors as in the 1P-B control. We then clustered this generated library using Starcode with L = 3, and applied a cumulative count cutoff at 99%.

#### Selection of barcodes by cumulative count cutoff

The representatives of the clusters were cut off using a cumulative count criterion. Unless otherwise noted, the barcodes whose collective counts accounted for 99% of all counts were kept, and the rest discarded. Applying this threshold removed likely artifacts, including barcodes whose length was significantly more than 12 nt (Fig 1).

## Supporting information

S1 Table 10-plasmid controls preparation. (DOCX)

S2 Table Staggered indexing primers. (DOCX)

S1 Fig Schema representing the steps involved in the construction of the plasmid library and the barcoded muPyV genomes used for the subsequent generation of the virus library. Figure partly created with BioRender.com and SnapGene software (from Insightful Science; available at snapgene.com).
(TIF)

S2 Fig Schema representing the steps involved in the generation of the virus library. Figure partly created with BioRender.com.
(TIF)

S3 Fig Schema representing the enrichment and indexing PCR primers binding site in the region surrounding the barcode in a muPyV barcoded genome. Figure partly created with SnapGene software (from Insightful Science; available at snapgene.com).
(TIF)

S4 Fig Illumina sequencing reads from 10-plasmid controls using different clustering distances. The y-axis depicts the barcode sequence; the x-axis shows the square root-transformed percentage of total read counts. The colored bars represent correctly recalled input barcodes. Gray bars represent the most common erroneous barcodes for each clustering parameter. Here we show the 10-plasmid controls 10P-B, 10P-C, 10P-E and 10P-F.
(TIFF)

S5 Fig Illumina sequencing reads from 10-plasmid controls comparing Starcode’s message-passing vs spherical algorithms for increasing L distances. The y-axis panels vary the Levenshtein distance parameter; the x-axis panels show the message-passing vs spherical results side-by-side. The bar lengths are the square root-transformed total read count percentages. The colored bars represent correctly recalled input barcodes. Gray bars represent the most common erroneous barcodes for each L distance across either algorithm. Here we show the 10-plasmid controls 10P-A, 10P-D, and 10P-G.
(TIFF)

S6 Fig Linearity plots of 10-plasmid controls with L3 clustering parameter. The log10 transformed x-axis show the copy number of plasmid inputs, the log10 transformed y-axis represents L3 clustered read counts. Linear regression trendlines are plotted in gray, with corresponding R^2^ values. Linearity in 10-plasmid controls 10P-B and 10P-F is shown.
(TIFF)
