# Supplementary material for: Interpreting and de-noising genetically engineered barcodes in a DNA virus
Source: PLoS Comput Biol. 2022 Nov 22;18(11):e1010131. doi: 10.1371/journal.pcbi.1010131 (PMC9725130; doi:10.1371/journal.pcbi.1010131)
Supplement: S1 Table — (DOCX) [file pcbi.1010131.s001.docx]

**Table S1. 10-plasmid controls preparation.**

| **Barcodes** | **10P-B** | | **10P-C** | | **10P-E** | | **10P-F** | |
| --- | --- | --- | --- | --- | --- | --- | --- | --- |
|  | **Plasmid copies** | **PCR cycles** | **Plasmid copies** | **PCR cycles** | **Plasmid copies** | **PCR cycles** | **Plasmid copies** | **PCR cycles** |
| TCACAGGGGTAA | 1.00E+04 | 32 | 1.00E+03 | 35 | 1.00E+04 | 29 | 1.00E+05 | 29 |
| ACAAGACCGGAA | 1.00E+04 |  | 1.00E+03 |  | 1.00E+04 |  | 1.00E+05 |  |
| ATATAGAGCTGT | 1.00E+02 |  | 1.00E+03 |  | 1.00E+04 |  | 1.00E+04 |  |
| ACATACCTGCTA | 1.00E+02 |  | 1.00E+01 |  | 1.00E+04 |  | 1.00E+04 |  |
| GTGTCAGGCACA | 1.00E+01 |  | 1.00E+01 |  | 1.00E+04 |  | 1.00E+03 |  |
| TGCCACTCTAGC | 1.00E+01 |  | 1.00E+01 |  | 1.00E+04 |  | 1.00E+03 |  |
| CTCGATTCACTC | 1.00E+01 |  | 1.00E+01 |  | 1.00E+04 |  | 1.00E+02 |  |
| GAACCCGTGGAA | 1.00E+01 |  | 1.00E+01 |  | 1.00E+04 |  | 1.00E+02 |  |
| CTGTATATTTTA | 1.00E+01 |  | 1.00E+01 |  | 1.00E+01 |  | 1.00E+01 |  |
| GAAACCATGACA | 1.00E+01 |  | 1.00E+01 |  | 1.00E+01 |  | 1.00E+01 |  |
