# Supplementary material for: Interpreting and de-noising genetically engineered barcodes in a DNA virus
Source: PLoS Comput Biol. 2022 Nov 22;18(11):e1010131. doi: 10.1371/journal.pcbi.1010131 (PMC9725130; doi:10.1371/journal.pcbi.1010131)
Supplement: S2 Table — (DOCX) [file pcbi.1010131.s002.docx]

**Table S2:** Staggered indexing primers.

|  | **Name** | **Index adapter** | **i5 or i7 index** | **Index adapter** | **Spacer** | **Annealing sequence** |
| --- | --- | --- | --- | --- | --- | --- |
| **i5 index forward primers** | >NGS_FwdIN2-i5-U6-F3-D501 | AATGATACGGCGACCACCGAGATCTACAC | TATAGCCT | ACACTCTTTCCCTACACGACGCTCTTCCGATCT | CAG | GACTGTATTTCCTGGAAATTAATGTT |
|  | >NGS_FwdIN2-i5-U6-F3-D502 | AATGATACGGCGACCACCGAGATCTACAC | ATAGAGGC | ACACTCTTTCCCTACACGACGCTCTTCCGATCT | CAG | GACTGTATTTCCTGGAAATTAATGTT |
|  | >NGS_FwdIN2-i5-U6-F2-D503 | AATGATACGGCGACCACCGAGATCTACAC | CCTATCCT | ACACTCTTTCCCTACACGACGCTCTTCCGATCT | G | GACTGTATTTCCTGGAAATTAATGTT |
|  | >NGS_FwdIN2-i5-U6-F2-D504 | AATGATACGGCGACCACCGAGATCTACAC | GGCTCTGA | ACACTCTTTCCCTACACGACGCTCTTCCGATCT | G | GACTGTATTTCCTGGAAATTAATGTT |
|  | >NGS_FwdIN2-i5-U6-F1-D505 | AATGATACGGCGACCACCGAGATCTACAC | AGGCGAAG | ACACTCTTTCCCTACACGACGCTCTTCCGATCT |  | GACTGTATTTCCTGGAAATTAATGTT |
|  | >NGS_FwdIN2-i5-U6-F1-D506 | AATGATACGGCGACCACCGAGATCTACAC | TAATCTTA | ACACTCTTTCCCTACACGACGCTCTTCCGATCT |  | GACTGTATTTCCTGGAAATTAATGTT |
|  | >NGS_FwdIN2-i5-U6-F4-D507 | AATGATACGGCGACCACCGAGATCTACAC | CAGGACGT | ACACTCTTTCCCTACACGACGCTCTTCCGATCT | GCAC | GACTGTATTTCCTGGAAATTAATGTT |
|  | >NGS_FwdIN2-i5-U6-F4-D508 | AATGATACGGCGACCACCGAGATCTACAC | GTACTGAC | ACACTCTTTCCCTACACGACGCTCTTCCGATCT | GCAC | GACTGTATTTCCTGGAAATTAATGTT |
|  | >NGS_FwdIN2-i5-U6-F5-D509 | AATGATACGGCGACCACCGAGATCTACAC | TTCGGATG | ACACTCTTTCCCTACACGACGCTCTTCCGATCT | AGCAC | GACTGTATTTCCTGGAAATTAATGTT |
|  | >NGS_FwdIN2-i5-U6-F5-D510 | AATGATACGGCGACCACCGAGATCTACAC | ACTCATAA | ACACTCTTTCCCTACACGACGCTCTTCCGATCT | AGCAC | GACTGTATTTCCTGGAAATTAATGTT |
| **i7 index reverse primers** | >NGS_Rev-i7-U6-R-D701 | CAAGCAGAAGACGGCATACGAGAT | ATTACTCG | GTGACTGGAGTTCAGACGTGTGCTCTTCCGATCT |  | GAATATAGCTGAATACACAGTTTATTC |
|  | >NGS_Rev-i7-U6-R-D702 | CAAGCAGAAGACGGCATACGAGAT | TCCGGAGA | GTGACTGGAGTTCAGACGTGTGCTCTTCCGATCT |  | GAATATAGCTGAATACACAGTTTATTC |
|  | >NGS_Rev-i7-U6-R-D703 | CAAGCAGAAGACGGCATACGAGAT | CGCTCATT | GTGACTGGAGTTCAGACGTGTGCTCTTCCGATCT |  | GAATATAGCTGAATACACAGTTTATTC |
|  | >NGS_Rev-i7-U6-R-D712 | CAAGCAGAAGACGGCATACGAGAT | AGCGATAG | GTGACTGGAGTTCAGACGTGTGCTCTTCCGATCT |  | GAATATAGCTGAATACACAGTTTATTC |
