# Supplementary figures and images for: Interpreting and de-noising genetically engineered barcodes in a DNA virus
Source: PLoS Comput Biol. 2022 Nov 22;18(11):e1010131. doi: 10.1371/journal.pcbi.1010131 (PMC9725130; doi:10.1371/journal.pcbi.1010131)

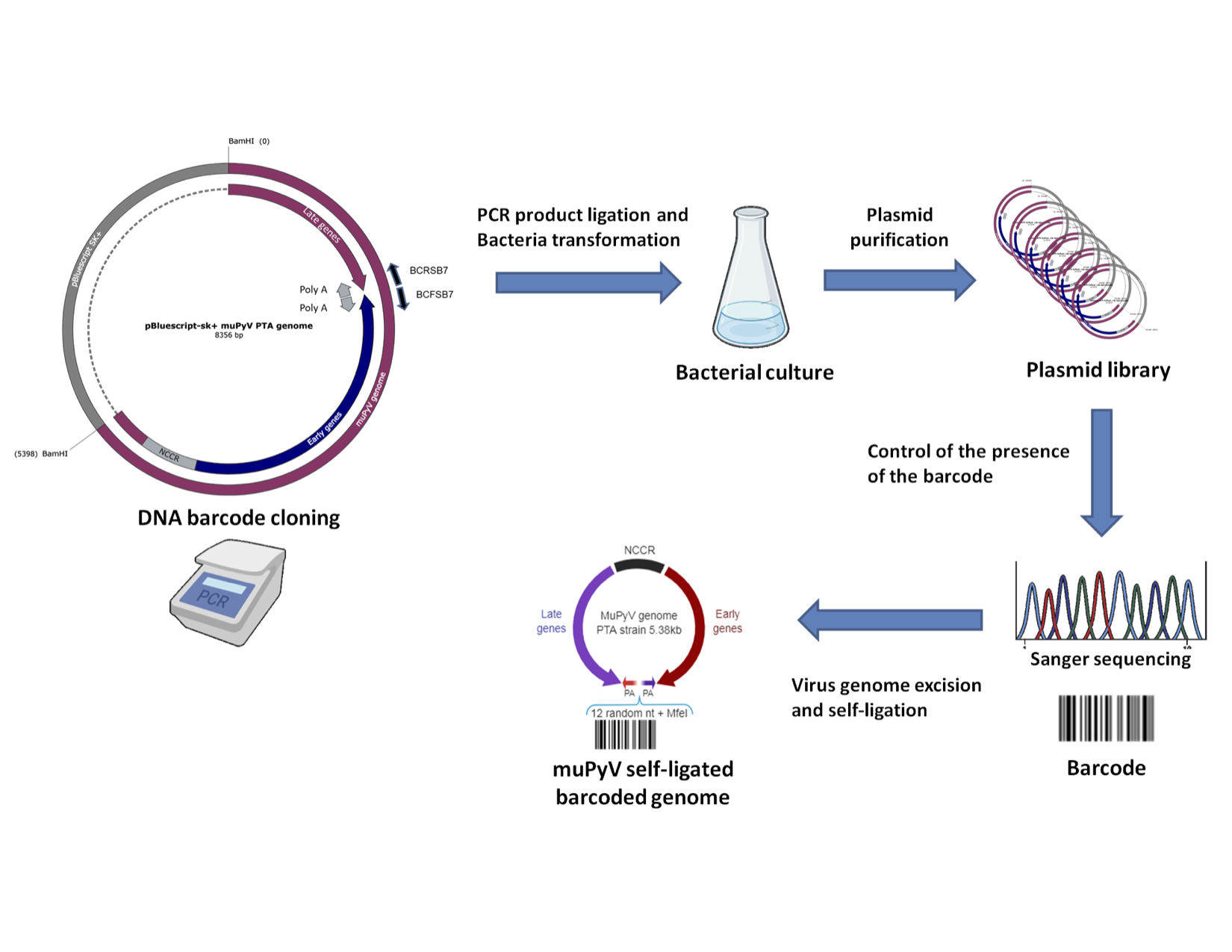

Supplement: S1 Fig — Figure partly created with BioRender.com and SnapGene software (from Insightful Science; available at snapgene.com). (TIF) [file pcbi.1010131.s003.tif]

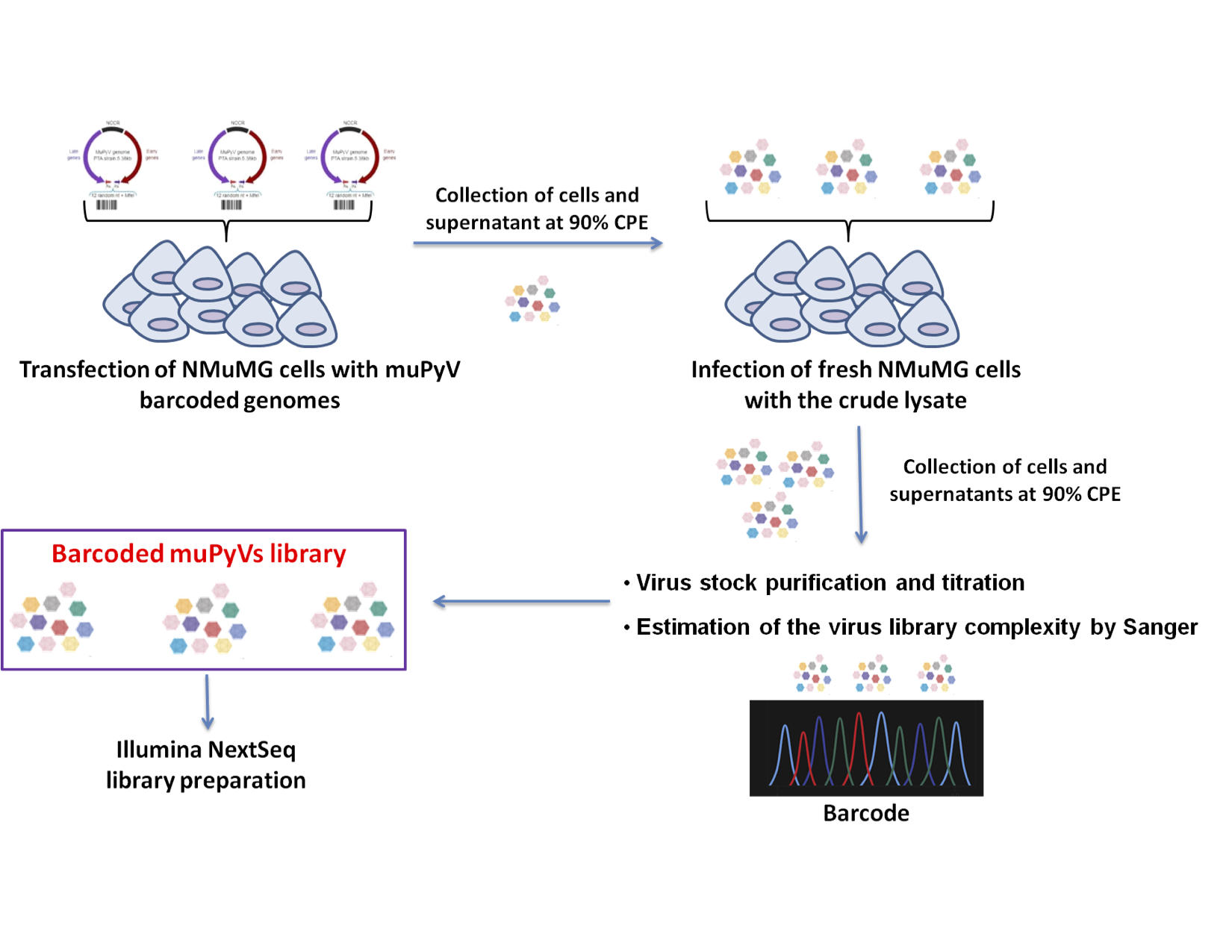

Supplement: S2 Fig — Figure partly created with BioRender.com. (TIF) [file pcbi.1010131.s004.tif]

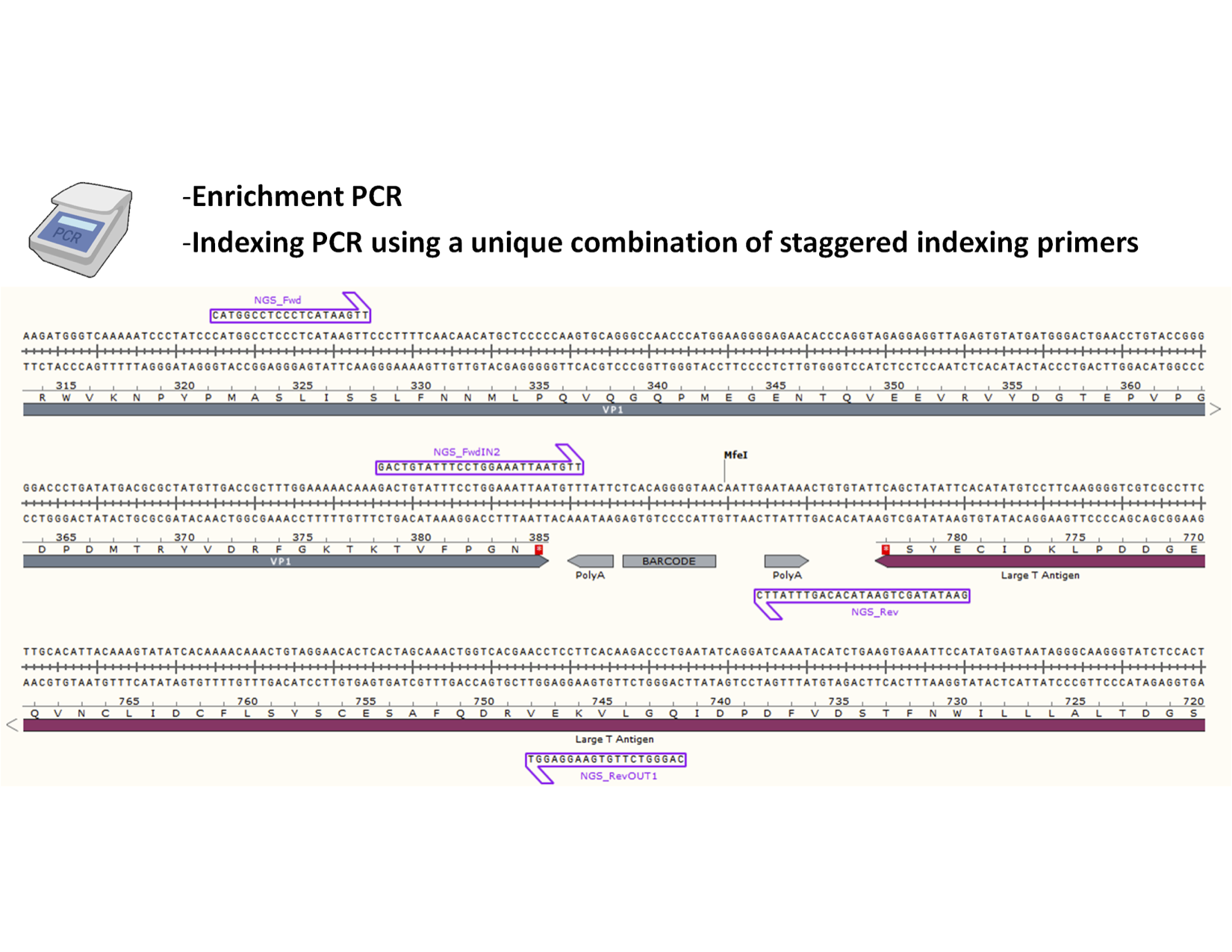

Supplement: S3 Fig — Figure partly created with SnapGene software (from Insightful Science; available at snapgene.com). (TIF) [file pcbi.1010131.s005.tif]

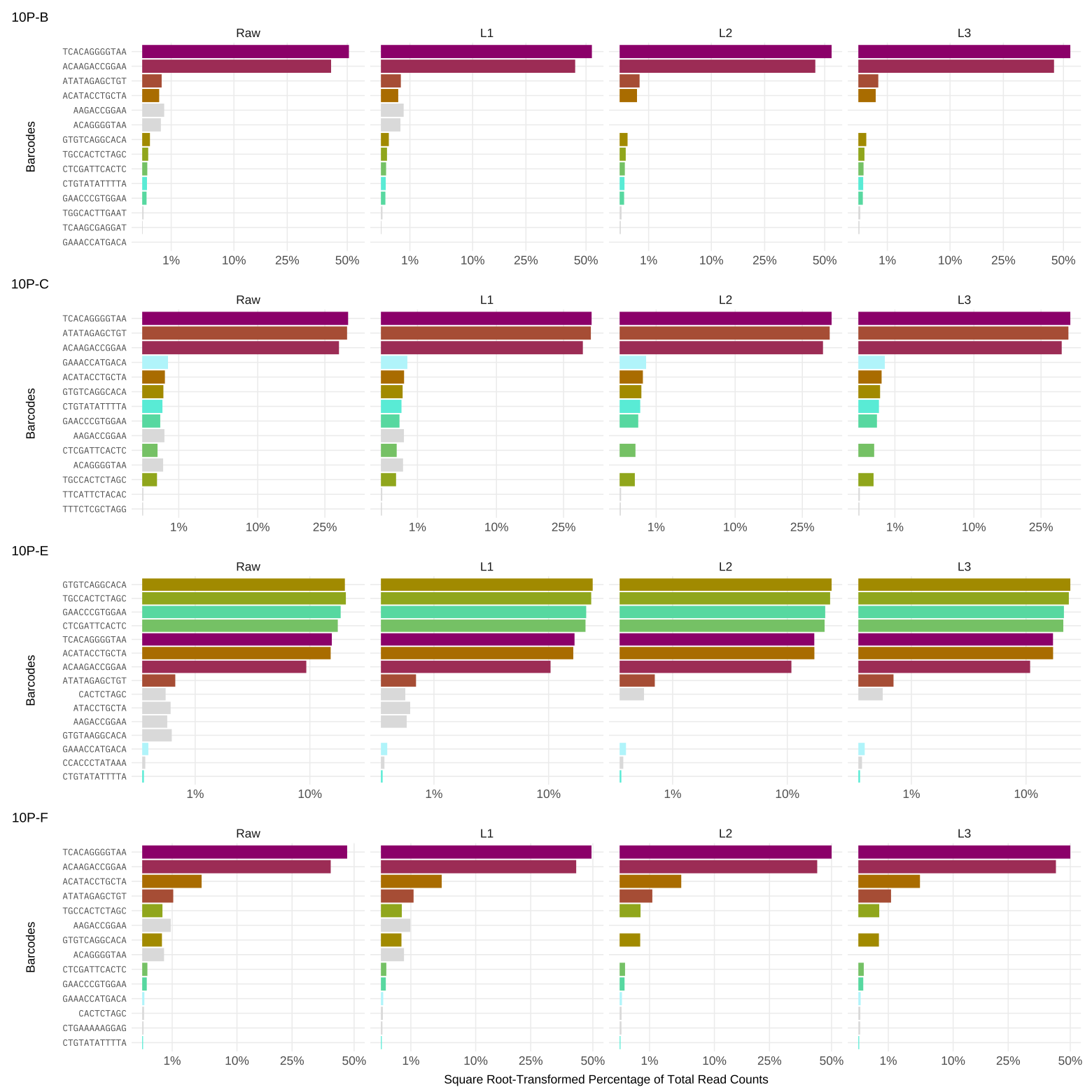

Supplement: S4 Fig — The y-axis depicts the barcode sequence; the x-axis shows the square root-transformed percentage of total read counts. The colored bars represent correctly recalled input barcodes. Gray bars represent the most common erroneous barcodes for each clustering parameter. Here we show the 10-plasmid controls 10P-B, 10P-C, 10P-E and 10P-F. (TIFF) [file pcbi.1010131.s006.tiff]

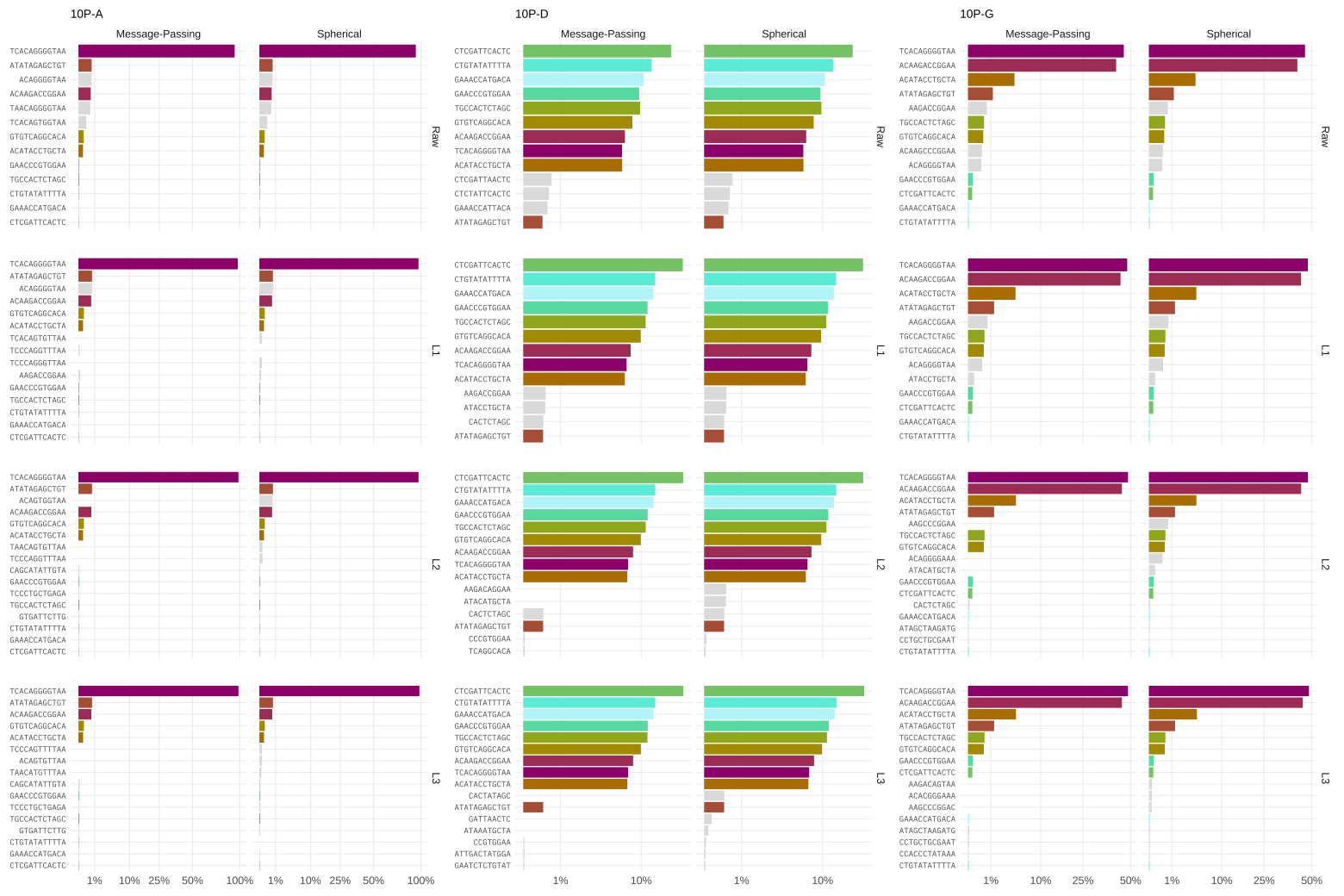

Supplement: S5 Fig — The y-axis panels vary the Levenshtein distance parameter; the x-axis panels show the message-passing vs spherical results side-by-side. The bar lengths are the square root-transformed total read count percentages. The colored bars represent correctly recalled input barcodes. Gray bars represent the most common erroneous barcodes for each L distance across either algorithm. Here we show the 10-plasmid controls 10P-A, 10P-D, and 10P-G. (TIFF) [file pcbi.1010131.s007.tiff]

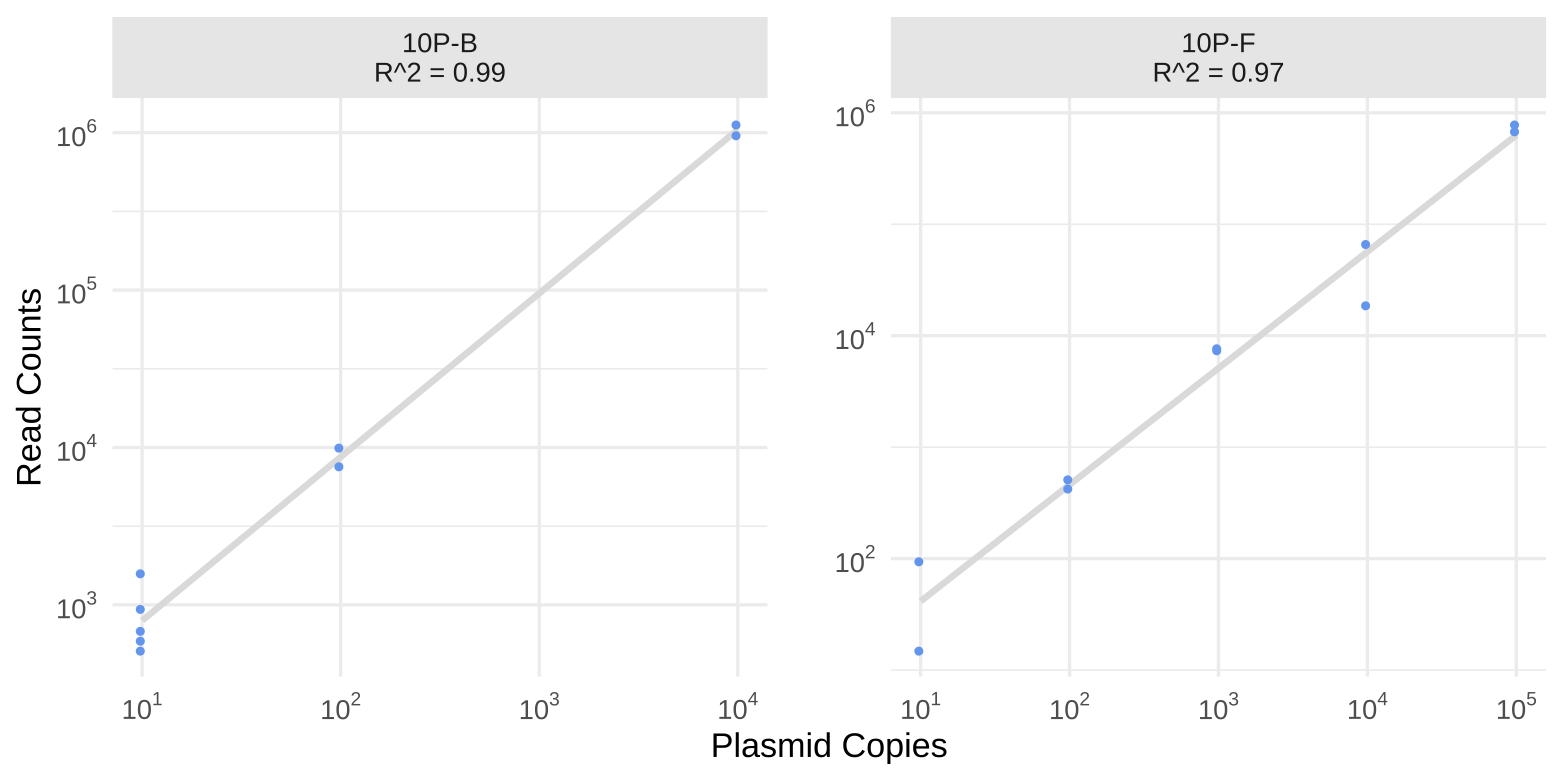

Supplement: S6 Fig — The log10 transformed x-axis show the copy number of plasmid inputs, the log10 transformed y-axis represents L3 clustered read counts. Linear regression trendlines are plotted in gray, with corresponding R2 values. Linearity in 10-plasmid controls 10P-B and 10P-F is shown. (TIFF) [file pcbi.1010131.s008.tiff]
