# Supplementary material for: Interpreting and de-noising genetically engineered barcodes in a DNA virus
Source: PLoS Comput Biol. 2022 Nov 22;18(11):e1010131. doi: 10.1371/journal.pcbi.1010131 (PMC9725130; doi:10.1371/journal.pcbi.1010131)
Supplement: Attachment — Submitted filename: Final939ResponseOct6CSDenoisingPlosCOMPesponse.pdf [file pcbi.1010131.s009.pdf]

Response to Reviews of Blois *et al.* "Interpreting and de-noising genetically engineered barcodes in a DNA virus"

Reviewers' comments in black, responses to reviewers in **blue**.

First, we are grateful for the comments, as they were on the mark and helped us appreciate how our message was not getting across. (The reviews helped us discover that we had not done some obvious analyses.) We either agree with all the review points or agree that they needed to be addressed in the manuscript. In fact, upon reading the comments, we stepped back and profoundly expanded the analysis (hence it was not a quick turnaround). So, the manuscript has undergone much revision including new analyses, simulations, and substantial modification of the text. The main message and conclusions have not changed, but the paper now offers far more justification, caveats, or context for what we conclude. We believe the product is now accessible to a broader swath of the PLoS Comp Bio readership and that these changes have much improved it.

Reviewer #1:

1. As the authors mention, the use of clustering algorithms for barcode identification has been used before. In fact, several clustering algorithms exist that are specifically developed for barcoded sequences (e.g. Orabi B et al. Bioinformatics. 2019. doi: 10.1093/bioinformatics/bty888 , Zhao L et al, Bioinformatics. 2018 doi: 10.1093/bioinformatics/btx655.) How does the message passing algorithm presented here compare to these methods? The authors mention a comparison to a single 'top-down' clustering approach briefly, but in order for the claim that the clustering method presented here is superior to hold, a more thorough comparison is needed.

Thank you for these references, we now cite them both. One of them is highly relevant and indeed offers a clustering method similar to ours ('Bartender' vs our use of 'Starcode'). That paper not only introduces Bartender but also compares it to Starcode and to some other clustering methods. Starcode performs approximately as well as Bartender, differing chiefly in the clustering of low-copy reads. But an important difference between our approach and the Bartender paper is our use of Levenshtein distances. We now make clearer that we are not making any claim that our approach is superior, acknowledging that the relative merits of each clustering protocol may depend on the nature of the errors in data as well as convenience in coding (see lines 333-42 and especially lines 342-4).

Further, we point out that typical 'top-down' clustering approaches are "greedy" algorithms that start with the most-common, or longest, sequence and then create a cluster of all sequences that are within a threshold distance of that initial sequence (see lines 303-23). For many applications these algorithms are useful, but not for barcodes where the lengths are in a very narrow range, and two barcodes within a threshold distance may actually be true barcodes. We contrast this approach to the message-passing algorithm by comparing Starcode's message-passing and spherical options, and now show in Figure S5 that the message-passing algorithm controls error in our 10-plasmid controls more efficiently than Starcode's implementation of a "top-down" spherical algorithm.

2. There are several sources of error in the process from engineering the plasmid to the creation of the virus library, yet all variation is lumped together as ''stemming from the Illumina library processing and

sequencing error". What is the theoretical expectation of variation at every step? And is it different than or similar to the observed variation? How many mutations are expected to occur due to replication in the plasmid? could this explain the higher-than-expected error rate? How much variation is expected to be introduced via virus replication, PCR, and sequencing? Does this match the observation that the number of PCR cycles has little impact on the observed variation?

The short answer is that we don't know for sure where the variation arises. We now offer a bioinformatics approach to mutation rates in the barcoded part of the plasmid, and that suggests the mutation rate is very low (see lines 154-57). Furthermore, Illumina is known to have problems when there isn't much nucleotide diversity in the first few-dozen bases; we attempted to avoid this, but we don't know. In response to this comment, our paper now delves into the matter more than it did before (for example, see lines 157-62), but it's not fully resolved. In any case, however, the problem is seen in other studies, and the bioinformatic solution doesn't obviously depend on the source of the error.

3. I am missing details on sequencing quality control. Were any reads discarded due to low quality scores? Were any quality filters applied to the raw reads and/or base calls?

Yikes! This omission was an obvious shortcoming of our study. We looked into it: using our controls, we found that setting various stringent thresholds for read quality (PHRED scores of 20 and 30) made some difference (1P controls) or no difference (10P controls) (newly modified Table 2, and text lines 273-5, (as well as other text). So, we are still left with clustering.

Minor points:
1. The manuscript contains many tables and figures, some of which could be combined or moved to supplemental information. **Agreed**.
a. table 1 belongs -- in my opinion -- in the supplement. **Done**.
b. table 2 can be removed and the values included into the sentence above ('Barcoded virus stocks gave rise to high titers of virus (wild type virus: , barcoded virus library:),...') **This table has been deleted as suggested**.
c. figures 2 and 3 have very small axis legends which are difficult to read **Fixed.**
d. figures 9/10/11 can be combined, or some of them moved to the supplement **As suggested, we combined Figures 10 and 11 (please see new Figure 10). However, we kept Figure 9 as a separate figure because it makes a different point showing high congruence of different technical replicates, which is a separate point from the congruence of libraries from different sources (plasmid, ligated virus genomes, virus) that went into of the final barcoded virus stock library.**
2. line 233: 20 / 21 of sequences were unique: what is the expectation from theory, or from your simulations? What is the probability of observing 20/21 unique sequences given your assumptions of random barcodes? **Ironically, we had an entire paragraph devoted to this problem (the 'birthday paradox') but cut it before submission. It is now back. The short answer is that 20/21 isn't very informative, but it does suggest that we did not have 5,000 equally abundant, unique barcodes. (See new lines 118-33).**
3. line 242/249: the exact number of different sequences are meaningless without the context of total number of reads, as more reads leads to the expectation of more distinct errors and thus more barcodes. A ratio (unique/total reads) would be useful for comparison **Fixed, (see lines 135-6; 142-3).**
4. line 250: 'this finding identifies ....'. This sentence is confusing: my initial interpretation was that large

variation is introduced in producing the virus library from the plasmid library, which is not supported by the aforementioned data. Please clarify. **In response to this, we have greatly expanded this section and changed the text, see lines 140-62.**

5. line 385: '...exhibits a shallow shoulder centered near 6000 barcodes ... ' This sentence is unclear: 6000 barcodes sounds like a count, but is a rank (using '6000th barcode' instead could improve clarity). **Fixed, see line 396-7.**

6. figure 6: what causes the sudden shift to 100% centroid percentage at the rank ~5000 barcode? **The clusters represented on the right consist of a single barcode of count 1, thus, the "centroid" (the barcode itself) trivially contributes 100% of the reads to the cluster. This is now added to the manuscript, lines 437.**

7. line 433: whole section. I don't understand why clusters were discarded based on frequency rather than length, if the goal is to weed out too-long sequences? It seems to me a length-based cutoff will achieve the same outcome, potentially keeping some very-low frequency true barcodes in place? **Given their existence in the library, we reasoned there must be some aberrant, low-count barcodes that remained in the library even after clustering, whether through the barcode extraction step, or some upstream anomaly in the library preparation. We have added text and re-wrote this section to better explain our rationale. Please see the entire paragraph starting at line 443.**

8. figure 8: what is the expected distribution from simulations? Does this match? **As requested, we conducted this simulation (see right panel of new Figure 8, gold) and the distributions match.**

9. line 640: you claim that using long enough barcodes is desirable, yet no evidence for what constitutes 'long enough' is provided. What is the effect of shorter barcodes? Why is 12 'long enough'? What decides what length is optimal? **The answer will depend on how many barcodes are created. There is no absolute answer, rather it's a matter of how much separation between barcodes is desirable. This point is now made clearer (see lines 566-9) and the overall manuscript should be much clearer on this point.**

Reviewer #2: Barcoded microbes have the potential to be profoundly important in tracking individual viral lineages within a complex and larger population. The authors have identified some of the problems with generating a barcoded microbe and have provided here a method to de-noise the barcodes by using pre-established clustering algorithms. Simply put, and universal to all next generation sequencing, this study seeks to determine what is likely real and likely PCR/sequencing error.

Suggestions to improve manuscript. I think it would be helpful to readers to show the data for raw, L1 and L2 in several other figures (or supplemental to the L3-only figures shown). What are the risks of over-clustering? I think one would be lower resolution of the population. Why can't all the data be retained even if clustered. So if barcode X has offspring Y, rather than just rolling it into X, somehow annotate this. **This comment gets at the essence of the entire paper, so our revision should be perceived as addressing it in many places and we now show and compare the raw data as requested, see Table 2 and Figures 2, 3 and 5. We also agree that a small loss of resolution is likely sacrificed in exchange for error control.**

The current proposed method still have no lower-limit. In Figure 5, there are thousands of barcodes with a count of 1. These are unlikely to be correct. I would suggest quantifying the level of input and use that value to set a hard floor in the sequence output. If 10K copies added to reaction, nothing less than 1/10K would be acceptable. **There are a couple of issues here. First, we now discard all barcodes with very**

low read counts – as suggested in this comment. Second, however, we face the problem (now more thoroughly acknowledged in the revision than in the first draft) that there is huge variation in true barcode abundance – spanning 100X. This means that the errors from the most common barcodes will be as abundant as the correct reads from the least common barcodes. Our 10P controls actually did a decent job of representing this variation, so one can see the problem with utilizing solely a strict threshold cutoff based on read counts (this is addressed in several parts of the manuscript, including lines 174, 186-92).

Also, and very important, how do the barcode proportions change by adding the errors back into the parent. If the errors are random, and very low in rank order, there might not be any proportional differences between this analysis and drawing a conservative cut-off. The next generation sequencing that I am familiar with just uses a cut-off determined by the shoulder and removes all data below that point. It would be very informative if you provided this comparison. **Based in part on this comment, we expand discussion of this possible resolution of barcode error and add new text (see line 182, lines 186-92). As noted in our response to the previous point, in the approach we converge upon, we do eliminate barcodes with very low reads, but a cut-off at 5,000 or 6,000 faces the problem identified above.**

Additionally, if multiple, low-dose, replicates were run on the stock and using 1/input values, "real" barcodes would show up again and again, while PCR errors would be limited and thereby either excluded or denoted as lower probability of being real. **This may be a possibility but we expect it would require a lot of sequencing and there is no *a priori* reason to believe it would work better than clustering. Related to this point, we now make it more clear that our analysis showed that our procedure of clustering and cutoff had very high overlap between the replicates of the viral library (see Figure 9 and lines 481-5).**

Overall, my view was that this analysis was specific to this one virus stock and not universally applicable. Things like 5-fold increase between parent and offspring, clustering distance of 3, cut-offs for long and short barcodes and how they are dealt with all seem to be just specific to stock. It would improve the manuscript immensely to discuss how one would do this if the stock distribution was much flatter than this stock, or if there were 50K barcodes rather than 5K. If the barcode was shorter etc. There are no robustness measures for this approach to help the average scientist trying to do this themselves. I think it reads like the preamble to a larger paper, but of course there isn't anything else. In my opinion, it can't be specific to the "study" without showing the study. Therefore, I would edit the text and provide a more general approach so it is more of a methods paper and provide simulations and estimates of what someone would do if trying to make this in another system.

We certainly did not intend this analysis to be specific to our study. This reviewer's comment helped inspire us to re-write large sections of text throughout the manuscript. We feel addressing this critique has improved its broad appeal. To reinforce our intended generality, we have modified the text in several places and now include a list of recommendations that can be applied broadly. In particular, please see the new section in lines 168-230 and the extensively modified Discussion section (especially lines 540-600).
