# Supplementary material for: Interpreting and de-noising genetically engineered barcodes in a DNA virus
Source: PLoS Comput Biol. 2022 Nov 22;18(11):e1010131. doi: 10.1371/journal.pcbi.1010131 (PMC9725130; doi:10.1371/journal.pcbi.1010131)
Supplement: Peer review history

# Peer review: Decision Letter 0

**DOI:** 10.1371/journal.pcbi.1010131.r001

**Authors:** Roland R Regoes, William Stafford Noble

1 Jul 2022

Dear Dr. Sullivan,

Thank you very much for submitting your manuscript "Interpreting and de-noising genetically engineered barcodes in a DNA virus" for consideration at PLOS Computational Biology.

As with all papers reviewed by the journal, your manuscript was reviewed by members of the editorial board and by several independent reviewers. In light of the reviews (below this email), we would like to invite the resubmission of a significantly-revised version that takes into account the reviewers' comments.

This work has been reviewed by two expert reviewers on barcoding viruses. They both acknowledge that work on the bioinformatics of barcodes is timely and valuable. However, they both criticized the lack of an in-depth discussion of previous work addressing these issues. They also raise issues about how different sources of errors have been dealt with (Reviewer#1 2. , and Reviewer #2 on "no lower-limit"). Reviewer #2 also finds that the work is only applicable to the stock used, and that the authors would need to make the method more generally applicable for publication in PLOS Comp Biol. We would strongly encourage you to expand your work according to this feedback.

We cannot make any decision about publication until we have seen the revised manuscript and your response to the reviewers' comments. Your revised manuscript is also likely to be sent to reviewers for further evaluation.

When you are ready to resubmit, please upload the following:

[1] A letter containing a detailed list of your responses to the review comments and a description of the changes you have made in the manuscript. Please note while forming your response, if your article is accepted, you may have the opportunity to make the peer review history publicly available. The record will include editor decision letters (with reviews) and your responses to reviewer comments. If eligible, we will contact you to opt in or out.

[2] Two versions of the revised manuscript: one with either highlights or tracked changes denoting where the text has been changed; the other a clean version (uploaded as the manuscript file).

Important additional instructions are given below your reviewer comments.

Please prepare and submit your revised manuscript within 60 days. If you anticipate any delay, please let us know the expected resubmission date by replying to this email. Please note that revised manuscripts received after the 60-day due date may require evaluation and peer review similar to newly submitted manuscripts.

Thank you again for your submission. We hope that our editorial process has been constructive so far, and we welcome your feedback at any time. Please don't hesitate to contact us if you have any questions or comments.

Sincerely,

Roland R Regoes

Associate Editor

PLOS Computational Biology

William Noble

Deputy Editor

PLOS Computational Biology

***********************

Reviewer's Responses to Questions

**Comments to the Authors:**

**Please note here if the review is uploaded as an attachment.**

Reviewer #1: In this paper, the authors present the process to create a barcoded virus library and a pipeline for downstream analysis to get around errors introduced in the library preparation and sequencing process. They suggest that to recover as many real barcodes as possible from a sequencing result that inherently will contain errors, a message passing clustering algorithm with Levenstein distance of 3 should be used.

The authors present a thorough analysis of all steps and results, comparing both Illumina and Sanger sequencing results from intermediate steps to get at real barcodes in the library.

With barcoding being used more and more in population biological studies, it is important to have robust data analysis pipelines and it is nice to see this part of the process get thoroughly vetted. Often, the process of extracting barcodes and dealing with errors in raw reads is overlooked in studies presenting results using barcoded populations.

While I find the work valuable and am impressed by the depth of the data analysis, there are some crucial elements missing for this work to fit in with the current literature.

1. As the authors mention, the use of clustering algorithms for barcode identification has been used before. In fact, several clustering algorithms exist that are specifically developed for barcoded sequences (e.g. Orabi B et al. Bioinformatics. 2019. doi: 10.1093/bioinformatics/bty888 , Zhao L et al, Bioinformatics. 2018 doi: 10.1093/bioinformatics/btx655. ) How does the message passing algorithm presented here compare to these methods? The authors mention a comparison to a single 'top-down' clustering approach briefly, but in order for the claim that the clustering method presented here is superior to hold, a more thorough comparison is needed.

2. There are several sources of error in the process from engineering the plasmid to the creation of the virus library, yet all variation is lumped together as ''stemming from the Illumina library processing and sequencing error". What is the theoretical expectation of variation at every step? And is it different than or similar to the observed variation? How many mutations are expected to occur due to replication in the plasmid? could this explain the higher-than-expected error rate? How much variation is expected to be introduced via virus replication, PCR, and sequencing? Does this match the observation that the number of PCR cycles has little impact on the observed variation?

3. I am missing details on sequencing quality control. Were any reads discarded due to low quality scores? Were any quality filters applied to the raw reads and/or base calls?

Minor points:

1. The manuscript contains many tables and figures, some of which could be combined or moved to supplemental information.

a. table 1 belongs -- in my opinion -- in the supplement

b. table 2 can be removed and the values included into the sentence above ('Barcoded virus stocks gave rise to high titers of virus (wild type virus: <x> , barcoded virus library: <y> ),...')

c. figures 2 and 3 have very small axis legends which are difficult to read

d. figures 9/10/11 can be combined, or some of them moved to the supplement

2. line 233: 20 / 21 of sequences were unique: what is the expectation from theory, or from your simulations? What is the probability of observing 20/21 unique sequences given your assumptions of random barcodes?

3. line 242/249: the exact number of different sequences are meaningless without the context of total number of reads, as more reads leads to the expectation of more distinct errors and thus more barcodes. A ratio (unique/total reads) would be useful for comparison

4. line 250: 'this finding identifies ....'. This sentence is confusing: my initial interpretation was that large variation is introduced in producing the virus library from the plasmid library, which is not supported by the aforementioned data. Please clarify

5. line 385: '...exhibits a shallow shoulder centered near 6000 barcodes ... ' This sentence is unclear: 6000 barcodes sounds like a count, but is a rank (using '6000th barcode' instead could improve clarity)

6. figure 6: what causes the sudden shift to 100% centroid percentage at the rank ~5000 barcode?

7. line 433: whole section. I don't understand why clusters were discarded based on frequency rather than length, if the goal is to weed out too-long sequences? It seems to me a length-based cutoff will achieve the same outcome, potentially keeping some very-low frequency true barcodes in place?

8. figure 8: what is the expected distribution from simulations? Does this match?

9. line 640: you claim that using long enough barcodes is desirable, yet no evidence for what constitutes 'long enough' is provided. What is the effect of shorter barcodes? Why is 12 'long enough'? What decides what length is optimal?</y></x>

Reviewer #2: Barcoded microbes have the potential to be profoundly important in tracking individual viral lineages within a complex and larger population. The authors have identified some of the problems with generating a barcoded microbe and have provided here a method to de-noise the barcodes by using pre-established clustering algorithms. Simply put, and universal to all next generation sequencing, this study seeks to determine what is likely real and likely PCR/sequencing error.

Suggestions to improve manuscript. I think it would be helpful to readers to show the data for raw, L1 and L2 in several other figures (or supplemental to the L3-only figures shown). What are the risks of over-clustering? I think one would be lower resolution of the population. Why can't all the data be retained even if clustered. So if barcode X has offspring Y, rather than just rolling it into X, somehow annotate this.

The current proposed method still have no lower-limit. In Figure 5, there are thousands of barcodes with a count of 1. These are unlikely to be correct. I would suggest quantifying the level of input and use that value to set a hard floor in the sequence output. If 10K copies added to reaction, nothing less than 1/10K would be acceptable.

Also, and very important, how do the barcode proportions change by adding the errors back into the parent. If the errors are random, and very low in rank order, there might not be any proportional differences between this analysis and drawing a conservative cut-off. The next generation sequencing that I am familiar with just uses a cut-off determined by the shoulder and removes all data below that point. It would be very informative if you provided this comparison.

Additionally, if multiple, low-dose, replicates were run on the stock and using 1/input values, "real" barcodes would show up again and again, while PCR errors would be limited and thereby either excluded or denoted as lower probability of being real.

Overall, my view was that this analysis was specific to this one virus stock and not universally applicable. Things like 5-fold increase between parent and offspring, clustering distance of 3, cut-offs for long and short barcodes and how they are dealt with all seem to be just specific to stock. It would improve the manuscript immensely to discuss how one would do this if the stock distribution was much flatter than this stock, or if there were 50K barcodes rather than 5K. If the barcode was shorter etc. There are no robustness measures for this approach to help the average scientist trying to do this themselves. I think it reads like the preamble to a larger paper, but of course there isn't anything else. In my opinion, it can't be specific to the "study" without showing the study. Therefore, I would edit the text and provide a more general approach so it is more of a methods paper and provide simulations and estimates of what someone would do if trying to make this in another system.

**********

**Have the authors made all data and (if applicable) computational code underlying the findings in their manuscript fully available?**

The PLOS Data policy requires authors to make all data and code underlying the findings described in their manuscript fully available without restriction, with rare exception (please refer to the Data Availability Statement in the manuscript PDF file). The data and code should be provided as part of the manuscript or its supporting information, or deposited to a public repository. For example, in addition to summary statistics, the data points behind means, medians and variance measures should be available. If there are restrictions on publicly sharing data or code —e.g. participant privacy or use of data from a third party—those must be specified.

Reviewer #1: Yes

Reviewer #2: Yes

**********

PLOS authors have the option to publish the peer review history of their article (what does this mean?). If published, this will include your full peer review and any attached files.

If you choose “no”, your identity will remain anonymous but your review may still be made public.

**Do you want your identity to be public for this peer review?** For information about this choice, including consent withdrawal, please see our Privacy Policy.

Reviewer #1: No

Reviewer #2: No

Figure Files:

While revising your submission, please upload your figure files to the Preflight Analysis and Conversion Engine (PACE) digital diagnostic tool, https://pacev2.apexcovantage.com. PACE helps ensure that figures meet PLOS requirements. To use PACE, you must first register as a user. Then, login and navigate to the UPLOAD tab, where you will find detailed instructions on how to use the tool. If you encounter any issues or have any questions when using PACE, please email us at figures@plos.org.

Data Requirements:

Please note that, as a condition of publication, PLOS' data policy requires that you make available all data used to draw the conclusions outlined in your manuscript. Data must be deposited in an appropriate repository, included within the body of the manuscript, or uploaded as supporting information. This includes all numerical values that were used to generate graphs, histograms etc.. For an example in PLOS Biology see here: http://www.plosbiology.org/article/info%3Adoi%2F10.1371%2Fjournal.pbio.1001908#s5.

Reproducibility:

To enhance the reproducibility of your results, we recommend that you deposit your laboratory protocols in protocols.io, where a protocol can be assigned its own identifier (DOI) such that it can be cited independently in the future. Additionally, PLOS ONE offers an option to publish peer-reviewed clinical study protocols. Read more information on sharing protocols at https://plos.org/protocols?utm_medium=editorial-email&utm_source=authorletters&utm_campaign=protocols

# Author comment: Author response to Decision Letter 0

**DOI:** 10.1371/journal.pcbi.1010131.r002

6 Oct 2022

Attachment Submitted filename: Final939ResponseOct6CSDenoisingPlosCOMPesponse.pdf Click here for additional data file.

# Peer review: Decision Letter 1

**DOI:** 10.1371/journal.pcbi.1010131.r003

**Authors:** Roland R Regoes, William Stafford Noble

8 Nov 2022

Dear Dr. Sullivan,

We are pleased to inform you that your manuscript 'Interpreting and de-noising genetically engineered barcodes in a DNA virus' has been provisionally accepted for publication in PLOS Computational Biology.

Before your manuscript can be formally accepted you will need to complete some formatting changes, which you will receive in a follow up email. A member of our team will be in touch with a set of requests.

Please note that your manuscript will not be scheduled for publication until you have made the required changes, so a swift response is appreciated.

IMPORTANT: The editorial review process is now complete. PLOS will only permit corrections to spelling, formatting or significant scientific errors from this point onwards. Requests for major changes, or any which affect the scientific understanding of your work, will cause delays to the publication date of your manuscript.

Should you, your institution's press office or the journal office choose to press release your paper, you will automatically be opted out of early publication. We ask that you notify us now if you or your institution is planning to press release the article. All press must be co-ordinated with PLOS.

Thank you again for supporting Open Access publishing; we are looking forward to publishing your work in PLOS Computational Biology. 

Best regards,

Roland R Regoes

Academic Editor

PLOS Computational Biology

William Noble

Section Editor

PLOS Computational Biology

***********************************************************

Reviewer's Responses to Questions

**Comments to the Authors:**

**Please note here if the review is uploaded as an attachment.**

Reviewer #1: The authors have provided a thoroughly revised manuscript. The revisions address all the issues raised and provide a much clearer picture of the experiments and analyses performed. The resulting paper is nuanced, very readable and valuable for any future studies involving barcoded populations.

I have no further comments.

Reviewer #2: I think the authors did a fine job correcting and updating the manuscript. They have responsibly responded to each question or comment.

**********

**Have the authors made all data and (if applicable) computational code underlying the findings in their manuscript fully available?**

The PLOS Data policy requires authors to make all data and code underlying the findings described in their manuscript fully available without restriction, with rare exception (please refer to the Data Availability Statement in the manuscript PDF file). The data and code should be provided as part of the manuscript or its supporting information, or deposited to a public repository. For example, in addition to summary statistics, the data points behind means, medians and variance measures should be available. If there are restrictions on publicly sharing data or code —e.g. participant privacy or use of data from a third party—those must be specified.

Reviewer #1: Yes

Reviewer #2: Yes

**********

PLOS authors have the option to publish the peer review history of their article (what does this mean?). If published, this will include your full peer review and any attached files.

If you choose “no”, your identity will remain anonymous but your review may still be made public.

**Do you want your identity to be public for this peer review?** For information about this choice, including consent withdrawal, please see our Privacy Policy.

Reviewer #1: No

Reviewer #2: No

# Editor report: Acceptance letter

**DOI:** 10.1371/journal.pcbi.1010131.r004

**Authors:** Roland R Regoes, William Stafford Noble

15 Nov 2022

PCOMPBIOL-D-22-00644R1 

Interpreting and de-noising genetically engineered barcodes in a DNA virus

Dear Dr Sullivan,

I am pleased to inform you that your manuscript has been formally accepted for publication in PLOS Computational Biology. Your manuscript is now with our production department and you will be notified of the publication date in due course.

The corresponding author will soon be receiving a typeset proof for review, to ensure errors have not been introduced during production. Please review the PDF proof of your manuscript carefully, as this is the last chance to correct any errors. Please note that major changes, or those which affect the scientific understanding of the work, will likely cause delays to the publication date of your manuscript. 

Soon after your final files are uploaded, unless you have opted out, the early version of your manuscript will be published online. The date of the early version will be your article's publication date. The final article will be published to the same URL, and all versions of the paper will be accessible to readers.

Thank you again for supporting PLOS Computational Biology and open-access publishing. We are looking forward to publishing your work! 

With kind regards,

Zsofia Freund

PLOS Computational Biology | Carlyle House, Carlyle Road, Cambridge CB4 3DN | United Kingdom ploscompbiol@plos.org | Phone +44 (0) 1223-442824 | ploscompbiol.org | @PLOSCompBiol
